# Strengths-Based Programs for Youth at Risk for Toxic Stress: A Scoping Review of Programs Targeting Mental Health, Substance Use, Parenting Skills, and Family Functioning

**DOI:** 10.1177/15248380251326902

**Published:** 2025-03-29

**Authors:** Afsaneh Saghafi, Sarah M. Rodrigues, Jayla Aldridge, Maruko Myint, Donna Balsam, Nayeli Inzunza, Julissa Hernandez, Stephen L. Clancy, Luis Monreal-Duarte, Dawn T. Bounds

**Affiliations:** 1 University of California, Irvine, USA; 2 San Diego State University, CA, USA

**Keywords:** adverse childhood experiences, child maltreatment, mental health effects, parenting

## Abstract

This scoping review explores and describes recent strengths-based programs for use among youth (ages 12–24) at risk for toxic stress that target mental health (MH) or substance use (SU) outcomes through improving family functioning (FF) and/or parenting skills (PS). Following Preferred Reporting Items for Systematic Reviews and Meta-Analyses Extension for Scoping Reviews guidelines, seven databases were searched for peer-reviewed articles published between 2018 and 2023. Inclusion criteria included articles describing programs delivered to both youth and caregivers that targeted MH or SU outcomes through improving FF and/or PS. Thirty-three articles describing 33 programs were identified, 25 of which were research studies. Programs predominantly employed behavioral frameworks with varied duration and caregiver involvement. Most targeted MH outcomes, with fewer addressing SU. Positive outcomes included reductions in youth depression and anxiety, and improvements in emotional regulation and FF. Gaps identified included a majority of studies conducted in high-income countries, a prevalence of non-experimental designs, and a lack of standardized outcome measures. Results highlight the potential of family-centered, relational health-based interventions in promoting youth resilience and identify a need for more rigorous evaluations, culturally-responsive interventions, and increased research in low- and middle-income countries. This review underscores the promise of these interventions while revealing significant areas for future research to enhance effectiveness and applicability among adversity-impacted youth.

## Introduction

Prolonged exposure to adversity and/or trauma (i.e., toxic stress) during childhood (0–17 years) may negatively impact short- and long-term health and social outcomes (Felitti et al., 1998; Hughes et al., 2017; Nelson et al., 2020; Shonkoff & Garner, 2012). The protective potential of positive relationships to mitigate or buffer these negative sequelae has been well-established through decades of research on resilience and positive youth development (Garbarino, 1991; Ungar, 2006; Werner & Smith, 1982). Building on this established evidence base, in 2021, the American Academy of Pediatrics released a policy statement on childhood toxic stress, which endorsed a renewed focus on relational health, focusing on safe, stable, and nurturing relationships with parents, caregivers, and/or other supportive adults (herein referred to as caregivers) to buffer childhood adversity and build resilience. Relational health refers to the capacity to develop and maintain stable, supportive interpersonal relationships that promote emotional well-being and resilience in the face of stress (Garner et al., 2021). This statement called for multi-generational approaches to build resilience and connection, and supporting caregivers to provide the safe, stable, and nurturing relationships that children need (Garner et al., 2021). Strengths-based programs that target familial relational health *along with* positive child health outcomes represent a comprehensive approach aligned with this renewed focus. However, the literature to date has focused on these approaches among young children, with less attention given to utilizing relational health approaches among adolescents and youth in transition (ages 12–24) (herein referred to as youth). Adolescence represents a developmental period characterized by significant biological changes (e.g., puberty, altered sleep patterns), cognitive maturation, and shifting social dynamics (e.g., peer relationships, family role transitions) (Lukoševičiūtė-Barauskienė et al., 2023). Given youth susceptibility to mental health (MH) symptoms (including depression, anxiety, post-traumatic stress symptoms, and emotional dysregulation) and risky behaviors (e.g., substance use (SU) and unprotected sex), the development of self-regulatory skills alongside supportive relationships becomes crucial. Youth need both external support through caregiver scaffolding and internal capacities to manage emotions and stress effectively. Therefore, relational approaches, such as facilitating youth–caregiver co-regulation, may be critically important during this developmental period (Murray et al., 2019).

In this review, we define adverse childhood experiences (ACEs) as potentially traumatic events that occur during childhood, which may negatively impact long-term health, ranging from childhood neglect and maltreatment to household dysfunction (e.g., domestic violence, parental separation/divorce, mental illness, SU, and incarceration) and community-level exposures (e.g., community violence, discrimination, housing instability, and food insecurity) (Center for Disease Control and Prevention, 2021; Felitti et al., 1998; Koita et al., 2018). Within this broader context, trauma refers to “an event, series of events, or set of circumstances experienced by an individual as physically or emotionally harmful or life-threatening and that has lasting adverse effects on the individual’s functioning and mental, physical, emotional, or spiritual well-being” (Substance Abuse and Mental Health Services Administration, 2014). Unbuffered chronic exposure to ACEs and/or trauma (herein referred to as adversity) may lead to toxic stress (Thakur et al., 2020). It is important to distinguish toxic stress from other types of stress responses: positive stress involves brief increases in heart rate and mild elevations in stress hormone levels and is considered a normal and essential part of healthy development; tolerable stress activates the body’s alert systems to a greater degree but is buffered by supportive relationships and limited in duration, allowing the brain and organs to recover. In contrast, toxic stress is the repeated and/or prolonged activation of stress response systems that may occur in the absence of buffering supportive relationships, and which may cause disruptions in brain circuitry and other biological systems (Shonkoff & Garner, 2012). When toxic stress responses occur during sensitive developmental periods, they can lead to long-term structural and functional changes in multiple biological systems. These changes include alterations in brain architecture (particularly in regions involved in the stress response, emotion regulation, and executive functioning), dysregulation of the hypothalamic–pituitary–adrenal axis, changes in immune system functioning, and epigenetic modifications (McCullough & Mathura, 2019; McEwen, 2017). Such neurobiological and physiologic dysregulations may lead to lifelong physical and/or MH problems (Shonkoff et al., 2021). Among youth, positive graded associations have been found between exposure to adversity and negative MH and SU outcomes (Bomysoad & Francis, 2020; Duke, 2018; Swedo et al., 2020). A recent literature review found that cumulative exposure to adversity raised adolescent risk for SU, including initiation, prevalence, frequency, and changes in use. That risk was found to be moderated or mediated by genetic, intrapersonal, and interpersonal factors, including supportive adult–child relationships (Hoffmann & Jones, 2022).

The purpose of this scoping review is to explore and describe available strengths-based interventions, services, or programs (herein referred to collectively as programs)—those that emphasize identifying and building upon existing family and youth capabilities, fostering resilience, and promoting positive development rather than solely focusing on problem remediation—for use among youth at risk for toxic stress due to prolonged or chronic exposure to adversity and/or having an adversity-related condition (i.e., adversity-impacted youth). This emphasis on chronicity is crucial, as it is the persistent, recurring nature of exposure—rather than isolated stressful events—that most significantly impacts developmental outcomes. Furthermore, the risk for such chronic exposure is not equally distributed across populations. Youth from marginalized communities face disproportionate risk due to systemic factors, including poverty, racial discrimination, neighborhood violence, housing instability, limited access to resources, and inadequate social support systems (Bernard et al., 2021; Opara et al., 2021). These structural inequities often create conditions where multiple stressors compound and persist over time, particularly affecting youth from racial/ethnic minority backgrounds, low-income communities, and other marginalized groups. Understanding these systemic contributors to toxic stress exposure is crucial when examining interventions aimed at supporting adversity-impacted youth. Chronic exposure to adversity and toxic stress can lead to profound impacts on youth MH and behavior, including increased risk for depression, anxiety, post-traumatic stress disorder (PTSD), and SU disorders (Bomysoad & Francis, 2020; Duke, 2018; Hughes et al., 2017; Swedo et al., 2020). These MH challenges often cascade into broader functional impairments, such as decreased academic performance, school dropout, difficulties with peer relationships, and reduced educational attainment. Toxic stress can also disrupt executive functioning and emotional regulation through alterations in brain architecture and stress response systems (McCullough & Mathura, 2019; McEwen, 2017; Ortiz et al., 2022), creating barriers to academic success and healthy social development (Shonkoff et al., 2021). Interventions designed for marginalized youth, who may be disproportionately exposed to chronic adversity and impacted by toxic stress, must be culturally responsive (i.e., infusion of cultural values, tailoring according to cultural norms; Banks et al., 2023; Bounds et al., 2023) and contextually relevant (i.e., compatibility with local context; Bounds et al., 2020, 2022; Parra-Cardona et al., 2021) to be effective. For this review, we assumed that youth experiencing chronic adversity, including chronic illness, or trauma-related symptoms were at risk for toxic stress, even when studies did not explicitly measure toxic stress responses. The research question guiding this scoping review is: *What recent strengths-based programs exist for youth at risk for experiencing toxic stress that target youth MH or SU outcomes through improving family functioning (FF) and/or parenting skills (PS)?* Results may help guide providers working with adversity-impacted youth and their families and may identify gaps and key areas of focus for future research.

## Methods

### Design

A scoping review methodology was used (Arksey & O’Malley, 2005) to explore and describe the extent, range, and nature of available strength-based programs for use among youth at risk for experiencing toxic stress and that target FF or PS along with youth MH or SU outcomes. The Preferred Reporting Items for Systematic Reviews and Meta-Analyses Extension for Scoping Reviews checklist (Tricco et al., 2018) was followed to report the results of this scoping review.

### Search Strategy

Comprehensive literature searches were performed by a health sciences librarian among seven academic online databases: PubMed (U.S. National Library of Medicine/NCBI), Medline Complete (EBSCOhost), CINAHL Complete (EBSCOhost), PsycINFO (ProQuest), the Web of Science Core Collection (Clarivate), Scopus (Elsevier), and the Cochrane Library. Initial searches were executed on August 11 and 12, 2021. Updates were performed on December 13, 2022, February 25, 2023, and January 19 and 30, 2024. The search results were exported from each database and imported into the Covidence systematic review software (2024) screening and data extraction tool. The preliminary and final search strategies were collectively developed by the scoping review team, which included a health sciences librarian.

The search strategies used keywords and subject headings, which were supported by the respective databases, with specific field designations added to broader keywords as needed. A complete listing of search terms is provided in Supplementary Material, Appendix A.

### Study Selection and Data Extraction Procedures

Database search results were initially limited to peer-reviewed articles published in English between 2000 and 2024; conference proceedings, book chapters, dissertations, and these were excluded. Following the import of search results into Covidence, inclusion and exclusion criteria were applied. Articles were initially included if they (a) described the delivery of an intervention, service, and/or program, (b) delivered the intervention, service, and/or program to both a youth *and* a caregiver, parent, or other supportive adult, and (c) included youth between the ages of 12 and 24 years old. While these criteria captured interventions potentially relevant to toxic stress, it is acknowledged that not all included studies explicitly used the term “toxic stress” or measured toxic stress responses. Studies were included if they addressed chronic or severe adversity, trauma exposure, or their behavioral/emotional sequelae, based on the assumption that these populations were at risk for toxic stress due to their exposure histories and symptom presentations.

Exclusion criteria were applied in a stepwise manner. In the first step, articles were excluded if they (a) did not describe the delivery of an intervention, service, and/or program; (b) described the delivery of an intervention, service, and/or program to either a youth *or* a caregiver (a parent or other supportive adult); or (c) included only youth under 12 years old. In the second step, articles were excluded if they did not focus on mitigating youth trauma, stress, dysregulation, or on promoting youth self-care/management, self-efficacy, resilience, or self-regulation/co-regulation (behavioral, emotional, and cognitive). The third step excluded articles that did not target youth MH) or SU outcomes *and* family outcomes (FF or PS). While we included studies from diverse cultural and contextual environments, our intention was not to assume generalizability across these different environments. Rather, we aimed to capture the full landscape of available programs while acknowledging that implementation effectiveness may vary significantly based on culture, local resources, and other environmental contexts. This inclusive approach allows us to identify patterns in program development while recognizing the need for careful consideration of cultural and contextual factors in program implementation. In the fourth step, while database search results were initially limited to peer-reviewed articles published between 2000 and 2024, an additional criterion was applied during full-text review to include only articles published in the last 5 years (2018–2023) to ensure that our review captured the most recently described programs in the literature. Updates to our search were performed in December 2022, February 2023, and January 2024 to maintain currency. Finally, in cases where multiple articles described the same program, only the most recently published article that provided a full and detailed description of the program was included in the final analysis.

A manual search was also conducted to mine articles and reference lists from excluded articles to identify any named programs that potentially met our inclusion criteria but may have been missed in our initial database searches. When such programs were identified, targeted searches for these specific program names were conducted in our original databases (PubMed, Medline Complete, CINAHL Complete, PsycINFO, Web of Science Core Collection, Scopus, and Cochrane Library) using the program name as the primary search term. These targeted searches followed the same inclusion/exclusion criteria as our main search strategy, ensuring consistent selection procedures across all identified programs. Data extraction captured program characteristics (theoretical frameworks, duration/frequency, caregiver involvement, session format, content, and program target outcomes) and study characteristics (methodological design, participant demographics, study outcomes, and measures) by using the Covidence data extraction tool. Each extraction was performed independently by two data extractors and any conflicts were resolved by the senior author (DTB). An Excel spreadsheet was used to manage the extracted data.

### Search Results

**Figure 1.**
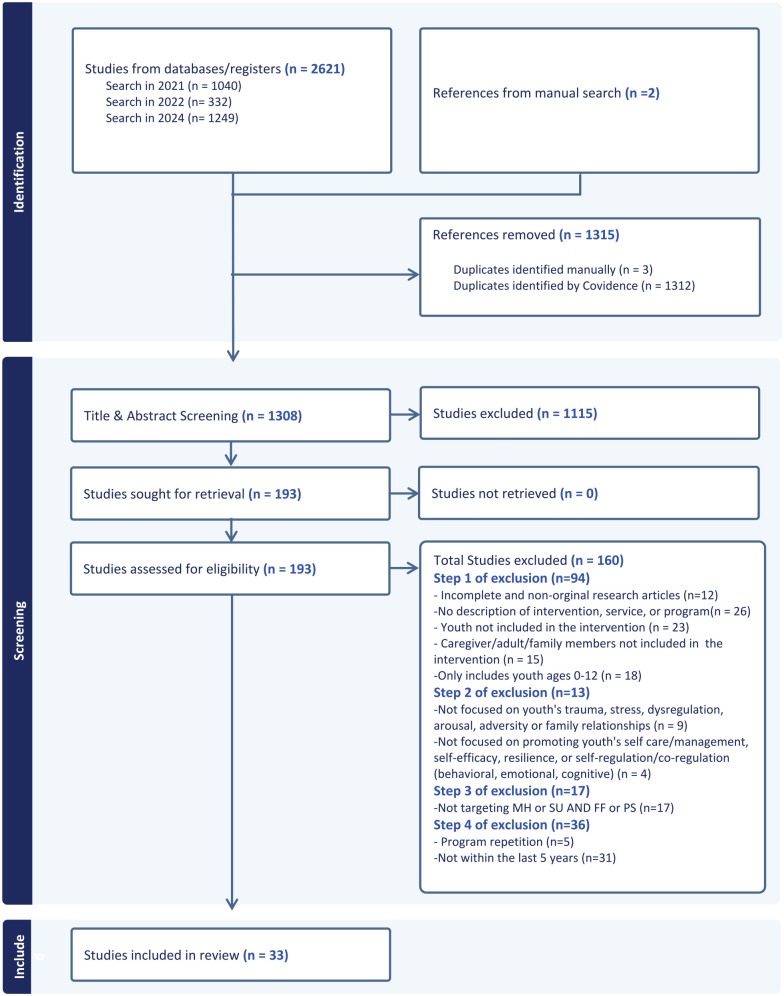
PRISMA diagram of the scoping review process. FF = family functioning; MH; mental health; PRISMA = Preferred Reporting Items for Systematic Reviews and Meta-Analyses; PS = parenting skills; SU = substance use.

The total number of articles added from initial and updated searches, including articles identified through targeted manual database searches from program mining of excluded articles, is presented in Figure 1. In total, 1,115 articles were excluded during title and abstract screening and an additional 160 articles were excluded during the full-text review in Covidence. In total, 33 programs were identified through 33 articles included in this scoping review.

## Results

In total, 33 articles describing 33 unique programs met the criteria for inclusion in this review. Articles were published between 2018 and 2023 and included 25 research studies and 8 descriptive articles. Included articles described 19 programs [2, 4, 5, 6, 7, 8, 10, 12, 13, 15, 16, 17, 18, 19, 20, 23, 24, 26, 29] delivered in the United States, three delivered in Sweden [21, 27, 31], two in Australia [30, 33] and the United Kingdom [11, 28], and one each in Switzerland [1], Italy [3], Canada [9], the Netherlands [14], Spain [22], Germany [25], and Africa [32]. A summary of the program and study characteristics is presented in Table 1 and Appendix B.

**Table 1.** Program Characteristics.

**Program Number**	**Program Name** (Author(s) and Year)	**Location**	**Program Target**			**Program Description**	**Frequency & Duration**
			**MH**	**SU**	**FF/PS**		
1	Family-based treatment (FBT- HT) (Besse-Flütsch et al., 2023)	Switzerland	✓		✓	Targets youth with anorexia eating disorders, A professional helps parents and youth increase behaviors that encourage the youth in feeding and reduce behaviors within the family circle that encourages the increase of symptoms of anorexia. Aims to decrease anorexia symptoms and improve parent–child relationships.	24, 60 to 90 min sessions over 12 weeks
2	Hybrid delivery of behavioral health screening and prevention intervention (Wijesekera et al., 2023)	United States	✓		✓	Targets pediatric heart transplant recipients and aims to improve the personal resilience and behavioral difficulties of youth and caregivers. It is a live-video telehealth behavioral program that included psychoeducation about medication adherence and post-traumatic stress related to their medical journey.	8 to 10 sessions over 3 months
3	Family Skills Program (Haar et al., 2023)	Italy	✓		✓	Targets parent & child dyads and focuses on caregiving approaches using empathy and warmth. Aim is to improve family relationships, child and caregiver mental health, and family resilience.	4, 120-min sessions over 4 weeks
4	System for Adult Growth and Emergence-Foundations (SAGE- F) Program (Taliercio et al., 2023)	United States	✓		✓	Targets young adults and aims to reduce depression, anxiety, non-suicidal self-injurious behaviors. Consists of mindfulness, emotional regulation, and an optional parental skills group that promotes positive parenting skills.	24, 150-min sessions over 6 weeks. Optional parental participation was 15 sessions over 15 weeks
5	Technology-enabled pediatric and family behavioral health Service (Loo et al., 2023)	United States	✓		✓	Focuses on targeting children/teens and parents with mental health issues through technology-focused interventions. This includes telehealth health coaching, therapy, and psychiatry.	4 to 6 coaching sessions; length of session not specified; over 4 to 6 weeks
6	Prevention-focused Program (Clauss et al., 2023)	United States	✓		✓	Targets youth from non-English speaking families and ethnically diverse backgrounds and aims to teach both youth and caregivers emotional regulation skills. Sessions led by licensed clinicians, with one of them being bicultural and bilingual.	10 sessions
7	Gentle, Interested, Validate, and Easy (GIVE) (Kaufman et al., 2023)	United States	✓		✓	Targets self-injuring adolescents and their mothers. Teaches interpersonal validation to promote emotional regulation among youth and parents and improve parent–child relationship.	1, 50-min session
8	Child and Family Traumatic Stress Intervention (CFTSI) (Stover et al., 2022)	United States	✓		✓	Targets children aged 7 and older who have experienced a recent traumatic event or disclosed abuse. It involves engaging the child and caregiver together to increase communication about the child’s trauma symptoms, teach coping strategies, and support the caregiver in responding effectively to the child’s posttraumatic stress symptoms (PTSS).	5 to 8 sessions
9	Building Resilience and Attachment in Vulnerable Adolescents (BRAVA) (Cloutier et al., 2022)	Canada	✓		✓	Aims to decrease suicidal behavior and increase family support for youth presenting with mild to moderation suicidal ideation. Utilizes CBT and problem solving to improve mood and/or emotional regulation.	6, 90-min modules over 6 weeks
10	The Family Strengthening Intervention for Refugees (FSI-R) (Neville et al., 2022)	United States	✓		✓	Targets family functioning and youth mental health problems among Somali Bantu and Bhutanese refugees. Aims to improve parent–child relationships, thereby lowering children’s risk of mental health issues.	10, 90-min sessions over 10 weeks
11	Parallel Parent–Child Mindfulness Intervention (Lu et al., 2022)	U.K.	✓		✓	Targets low-income Chinese families who are migrating from rural to urban areas to reduce parenting stress and improve family relationships and behavioral problems among the children.	8, 120-min sessions over 8 weeks for parents. 8, 15-to-20-min sessions over 8 weeks for children
12	Family Intervention Program (Brown et al., 2022)	United States	✓		✓	Targeted toward youth who experience adversity in Lebanon. It focuses on decreasing youth psychological distress, promoting positive mental health practices for both youth and caregivers, and positive parenting skills.	6, 90-min sessions over 6 weeks for families. 6, 30-min sessions over 6 weeks for parents. Followed by a booster session 1 month later
13	Familias con Orgullo (Families with Pride) Intervention (Lozano et al., 2022)	United States	✓	✓	✓	Aims to prevent/reduce substance use, sexual risk behaviors, and depressive symptoms among Latinx sexual minority youth. Goals are to increase family support and acceptance, improve communication, build adolescent resilience, and reduce stigma and discrimination to improve health outcomes.	3, 120-min sessions for adolescents. 7, 120-min sessions for parents. 4, 60-min sessions for families
14	MY mind (Siebelink et al., 2022)	Netherlands	✓		✓	Targets children with ADHD and their parents. Aims to improve youth self-control, distress tolerance, and parental mental health.	8, 90-min sessions over 8 weeks followed by 1 booster session 8 weeks later
15	Trust-Based Relational Intervention (Knight et al., 2021)	United States		✓	✓	Aims to prevent opioid and substance use among juvenile justice youth through an intervention that addresses relational trauma and promotes emotional regulation with the youth and caregiver.	Youth: 9, 45-min modules over 3 months. Caregiver: 60-min introductory module. 9, 90-min modules over 3 months. Youth+caregiver: 4, 60-min sessions over 3 months. Secondary part of intervention comprises either 4 or 2+ sessions with possibility for more sessions, as requested
16	RELAX (Regulating Emotions Like An Expert) intervention (Breaux et al., 2023)	United States	✓		✓	Target emotional regulation difficulties in youth with ADHD via multiple forms of media and delivery of educational material. Hopes to foster the caregiver-youth bond by meeting with their trainers individually and as a dyad.	8, 90-min sessions over 8 weeks
17	Community-Derived Mindfulness-Based Intervention (Li et al., 2021)	United States	✓		✓	Targets Latinx families that struggle through heightened stress and emotional dysregulation that initiates from the caregiver which follows to the child. Uses the concept of meditation and mindfulness to better the perceived social support of the child and family relationships.	7, 60-min sessions over 7 weeks
18	ProSAAF Protecting Strong (Lavner et al., 2021)	United States	✓		✓	Targets African American families and aims to improve parent–child relationships and prevent youth secondary stress from economic and family stress.	6, 120-min sessions over 6 weeks
19	Maximizing Adolescent Post- Secondary Success (MAPSS) (Kirby et al., 2021)	United States	✓		✓	Targets autistic youth anticipating high school graduation and their parents. Aims to guide parents in facilitating the development of independent living skills for adulthood in their autistic youth. Focused on goal setting, addressing challenges like executive functioning, and supporting the youth’s self-determination and positive autistic identity.	6, 90-min sessions over 6 weeks
20	A post-reunification service (Rushovich et al., 2021)	United States	✓		✓	Success Coaches work with families after a child is reunified with their family to help stabilize, build resiliency, and other protective factors within the family. Aims to promote positive parenting skills and prevent youth from re-entering care or returning to agent custody.	48, 60-min sessions over 2 years
21	Combined Parent Child Cognitive-Behavioral Therapy (CPC-CBT) (Thulin et al., 2020)	Sweden	✓		✓	Targets families with children who have experienced physical abuse. Aims to help parents replace corporal punishment with positive parenting strategies and reduce youth symptoms from traumatic experiences. Consists of separate parent and child groups that focus on psychoeducation, coping skills, family safety planning, and abuse clarification.	Not specified
22	Actions for the Treatment of Adolescent Personality (ATraPA) (Mayoral et al., 2020)	Spain	✓		✓	Improve the prognosis of adolescents diagnosed with BPD and prevent disorder development. Consists of 3 subprograms: AtraPA-TAI, Families on the Border and the Alternatives Group in the inpatient setting. Provides a safe space to learn adaptive strategies to cope with distress and promote emotional regulation skills for both youth and parents.	30, 120-min sessions over 24 weeks for skills group. 12, 120-min sessions over 24 weeks for parents
23	A smartphone app for self-monitoring of family functioning (Swendeman et al., 2020)	United States	✓		✓	Aims to promote self-regulation and improve family functioning through a smartphone app where families can record daily ecological momentary assessments about their daily health habits and parent–child interactions.	EMA/diary surveys prompted 4 times daily over 2 weeks
24	Risk Reduction Through Family Therapy (RRFT) (Danielson et al., 2020)	United States	✓	✓	✓	Targets adolescents who have experienced interpersonal violence experiences and other traumatic events and focuses on PTSD symptoms and substance use problems. Uses psychoeducation and multisystemic therapy to decrease substance use and PTSD symptom severity.	18, 40-to-90-min sessions
25	Strengthening Families Program (SFP) (Arnaud et al., 2020)	Germany		✓	✓	Aims to help prevent substance use in at-risk youth through mindfulness techniques. This is a subset of the strengthening families program focused on mindfulness and parents are also taught mindfulness techniques to promote parenting skills.	7, 180-min sessions over 7 weeks
26	Youth Advocate Programs (Silva et al., 2020)	United States	✓		✓	Targets youth and families in juvenile justice, behavioral health, child welfare, and developmental disabilities systems. Aims to promote resilience and protective factors against family dysfunction, mental health concerns, and substance use.	Not specified
27	Emotion regulation group skills training for adolescents and parents (Holmqvist Larsson et al., 2020)	Sweden	✓		✓	Targets adolescent and parent dyads in outpatient child and adolescent psychiatric clinics. Aims to improve emotional regulation skills for both youth and parents.	5, 120-min sessions over 15 months
28	Neuro-Physiological Psychotherapy (NPP) (McCullough & Mathura, 2019)	U.K.	✓		✓	Targets children who have experienced developmental trauma and present with multiple, clinically significant, and emotional, and behavioral difficulties. Uses psychotherapy, parental support and sensory interventions that focus on the primitive, limbic, and cortical brain to support and treat these youth.	An average of 47 sessions over 5.5 years (from assessment to retest)
29	Trauma Resilience and Recovery Program (Ridings et al., 2019)	United States	✓		✓	Targets pediatric traumatic injury patients and aims to promote emotional recovery in both youth and caregiver. Consists of emotional coping strategies and trauma-focused CBT.	60-to-90-min sessions over 8 to 12 weeks
30	Tuning Relationships with Music (Colegrove et al., 2019)	Australia	✓		✓	Targets parent-adolescent dyads with parents who have a history of trauma and are experiencing conflict. Uses music to improve emotional regulation, promote interpersonal functioning, and teach nonverbal communication to reduce parent–youth conflict.	8, 60-min sessions over 8 weeks
31	Intensive Contextual Treatment (ICT) (Wijana et al., 2018)	Sweden	✓		✓	Targets families with high symptom loads and adolescents with self-harm and suicidal thoughts. Aims to reduce youth symptoms and suicidal attempts and improve parental mental health.	Sessions at least twice a week over 3 months (more if needed); 6- and 12-month follow-ups
32	A Positive Cognitive Behavior Therapy Program (Van Der Westhuizen et al., 2018)	Africa	✓		✓	Targets adolescent–parent relationships and aims to promote personal resilience and improve psychological well-being. Teaches CBT techniques and exercise and measures HRV to track how the vagal tone changes in response to emotional regulation.	7 sessions over 7 weeks
33	The Ryde Child and Youth Mental Health Service (Gill et al., 2018)	Australia	✓		✓	Targets families and adolescents struggling with emotional regulation. Utilizes dialectical behavior therapy administered in a group format and includes an adolescent skills group focusing on emotion regulation, distress tolerance, interpersonal effectiveness and mindfulness, along with a concurrent parent group to enhance parents’ ability to support their adolescents.	Sessions (not defined) over 6 months

### Program Characteristics

#### Theoretical/Conceptual Frameworks and Approaches

Six overarching theoretical or conceptual frameworks and/or approaches were identified among the included programs. These included behavioral, resilience, developmental-based, social, systemic, and mindfulness frameworks. Seventeen programs utilized behavioral frameworks, such as dialectical behavioral therapy (DBT), acceptance and commitment therapy, and cognitive behavioral therapy (CBT) [4, 5, 7, 8, 9, 13, 18, 21, 22, 23, 24, 25, 27, 29, 31, 32, 33]. Four programs used mindfulness training [11, 14, 17, 25] and resiliency-based practices and training [2, 3, 20, 25]. Others used systemic frameworks, seven focusing on multisystemic therapy, wraparound, family relational and social networks [1, 5, 10, 12, 24, 26, 30]; four used social frameworks including social learning, biopsychosocial vulnerability, or social regulatory cycle [3, 6, 12, 16] and developmental frameworks with attachment-based and eco-developmental based orientations [9, 15, 19, 28]. Five programs incorporated more than one framework [3, 5, 12, 24, 25].

#### Duration and Frequency

Program duration and frequency varied among programs, with most programs including approximately 6 to 10 sessions over 6 to 12 weeks. The shortest program that included an FF intervention and employed a smartphone app for self-monitoring lasted for 2 weeks [23], and the Gentle, Interested, Validate, and Easy intervention was the briefest, which consisted of one session [7]. Programs with the longest duration included the Post-Reunification service, which spanned 48 sessions over 2 years [20], and the Neuro-Physiological Psychotherapy program, which included an average of 47 sessions over 5.5 years [28]. Table 1 provides details on program duration and frequency among included programs.

#### Caregiver Involvement

Caregiver involvement among programs also varied. Fifteen programs included legal guardians or primary caregivers [1, 3, 5, 6, 8, 9, 10, 12, 15, 16, 17, 20, 25, 26, 29], 14 included birth parents only [2, 4, 11, 14, 18, 19, 21, 22, 23, 24, 30, 31, 32, 33], 2 included foster parents or adoptive parents [13, 28], 1 included mothers only [7], and 1 included biological parents or foster parents [27].

#### Session Format

Session format among programs included 13 programs that used both combined (youth and caregiver) and individual (youth or caregiver) sessions [5, 6, 8, 12, 14, 15, 18, 21, 22, 26, 29, 31, 33], 11 that used combined sessions only [1, 2, 7, 13, 16, 19, 20, 27, 28, 30, 32], seven that used individual sessions only [4, 9, 10, 11, 17, 23, 24], one that used multi-family group sessions [25], and one that used a combination of multi-family group and individual (youth or caregiver) sessions [3].

#### Session Content

Session content varied among programs with most programs having multiple foci. Five programs provided psychoeducation combined with therapeutic support services (including emotional regulation skills training, trauma-focused interventions, and parent–youth relationship enhancement strategies) [27, 28, 29, 30, 33], four focused on enhancing communication and relationships [10, 18, 28, 32], three taught mindfulness and emotional regulation [14, 17, 22] and supported parenting and behavioral skills [3, 5, 19], and one program focused on building resilience and positive youth development [6].

#### Program Target Outcomes

Target outcomes among programs encompassed youth and/or caregiver MH promotion, youth SU prevention, and improving FF and/or PS. Thirty-one programs assessed MH outcomes among youth and/or caregivers; 19 of these primarily targeted improvements in behavioral regulation among youth and/or caregivers [2, 3, 6, 7, 8, 11 12, 17, 19, 20, 22, 23, 26, 27, 28, 29, 30, 32, 33], while others targeted reductions in youth and/or caregivers MH issues [5, 10], youth self-harm and suicidal ideation [9, 31], youth attention-deficit/hyperactivity disorder (ADHD) [14, 16], youth PTSD [21, 24], youth stress [18], youth depression [13], and youth anorexia [1]. Many programs focused on multiple MH outcomes in addition to a primary MH target. For example, one program [4] targeted symptoms of youth depression, anxiety, and non-suicidal self-injurious behaviors. Four programs assessed youth SU, which included SU reduction and prevention [13, 15, 24, 25]. Two programs targeted both MH and SU reduction/prevention among youth [13, 24].

All programs identified FF and/or PS as primary outcomes, including 12 programs that focused on family relationships [1, 3, 5, 7, 10, 11, 16, 17, 18, 23, 26, 32], 11 that focused on positive PS [2, 4, 6, 12, 14, 15, 20, 21, 22, 25, 29], five that focused on parental support and acceptance [9, 13, 19, 28, 33], three that focused on communication [8, 24, 27], and two that focused on reducing family conflict/challenging behaviors [30, 31]. Many programs focused on multiple FF outcomes in addition to a primary outcome.

## Research Studies on Included Programs

The 33 articles included in this review included eight descriptive papers and 25 research studies. The eight descriptive papers included detailed descriptions of program development, previous studies conducted to evaluate these programs, and/or conceptual models of program frameworks, and mechanisms. The 25 research studies included in this review are summarized below. Appendix B provides details on the 25 included research studies.

### Study Designs

Various methodological designs were employed among the 25 research studies. Fourteen used non-experimental designs, including nine pilot/feasibility studies [4, 8, 9, 17, 19, 21, 27, 33] and one each of the following: a non-randomized trial using a matched pair design [28], a case study [32], a longitudinal within-group repeated measures study [31], a secondary observational study [7], and a retrospective cohort study [5]. Five of the research studies were randomized controlled trials (RCTs). Five of the studies used mixed methods designs, all of which combined pre-and post-test paradigms with qualitative data collection [6, 10, 11, 18, 20]. Two studies were purely qualitative, including a post-intervention focus group study [13] and a feasibility study [23].

### Participant Characteristics

In total, 25 research studies were included, with sample sizes ranging from two [32] to 1190 families [8] (Appendix B). From these studies, representing a total of 3,954 youth who reported gender, 36.8% identified as male (*n* = 1456), 62.6% identified as female (*n* = 2478), and 0.22% reported other gender identities, which included transgender and queer nonconforming youth [4]; 0.4% of youth were not reported or missing. All studies included youth between the ages of 12–18 years; however, some studies also included younger youth (as young as 2 years old [5, 20]) and older youth (as old as 31 years [4]). Of the studies conducted in the United States, representing 2,923 youth participants, 36.1% were reported as Caucasian (*n* = 1,058), 26.6% as Black/African American (*n* = 777), 3.4% as mixed race (*n* = 100), 2.12% as Asian/Pacific Islander (*n* = 62), 0.17% American Indian/Alaskan Native (*n* = 5), 5% as other races/ethnicities (*n* = 152) including Somali Bantu and Bhutanese [10], and 26.3% were listed as other or not reported (*n* = 769). 26% of all participants were reported as Hispanic/Latino (*n* = 766). The rest of the studies were conducted outside the United States [9, 11, 14, 21, 27, 28, 30, 31, 32] and did not report race and ethnicity in their results or provided undistributed and/or ambiguous reporting on race and ethnicity.

### Study Outcomes

Study outcomes encompassed promoting youth and/or caregiver MH, preventing youth SU, and improving FF and/or PS.

#### Mental Health Outcomes

The 25 studies employed a wide range of validated MH assessment tools to evaluate MH outcomes and reported improvements in youth and/or caregiver MH outcomes across multiple areas (see Appendix B).

#### Depressive Symptoms

Ten studies reported significant reductions in depressive symptoms among youth or caregivers. Eight studies reported significant reductions in depressive symptoms among youth [4, 5, 6, 9, 10, 14, 31, 32], with one study reporting decreased depressive symptoms among LGBTQ youth specifically [13]; another reported significant improvement in youth ADHD and depressive symptoms [14]. Two studies reported significant reductions in caregivers’ depressive symptoms [2, 32]. One study found no statistically significant improvement in depressive symptoms despite a moderate effect size [33], and two others reported no changes in depressive symptoms following their interventions [21, 27] among youth. For detailed statistical findings on depressive symptom outcomes, see Appendix B.

#### Anxiety Symptoms

Eight studies reported significant reductions in youth anxiety symptoms [4, 5, 6, 9, 10, 14, 31, 32, 33]. Two studies found no significant changes in youth anxiety symptoms [21, 27].

#### Self-Injurious Behavior/Suicidal Ideation and PTSD/Trauma Symptoms

Four studies reported significant decreases in youth self-injurious behaviors and/or suicidal ideation [4, 7, 9, 31]. Three studies reported reductions in youth PTSD and/or trauma symptoms [8, 21, 24]. One study reported significant reductions in parents’ post-traumatic stress symptoms [2]. One study reported no significant difference in youth trauma symptoms [10].

#### Stress

Three studies reported decreased parenting stress among intervention group caregiver participants [5, 11, 17). Two studies each reported significant improvements in youth stress [14, 33] and a significant reduction in perceived stress among youth and caregivers [9, 31]. One study reported no significant change in parents’ coping and stress [19].

#### Overall Internalizing/Externalizing Symptoms

Three studies reported significant reductions in youth internalizing symptoms [5, 28, 31], while one of these also reported significant decreases in externalizing behaviors among youth participants [28]. One study reported that the family intervention buffered against the negative impact of financial strain on parenting and youth conduct problems indirectly in the intervention group [18].

#### Emotional Regulation

Eight studies reported improvements in various aspects of emotional regulation among youth participants [4, 7, 16, 17, 27, 28, 30, 33]. Two studies reported improved emotion regulation among caregivers during challenging parenting situations [6, 23], and one study reported small improvements in caregiver emotion dysregulation [16].

#### Psychological well-Being/functioning

Among youth, one study reported significant improvements in overall psychosocial functioning [5], one reported enhancement in cognitive reappraisal [31], and another reported an increase in positive attribution bias among youth [6]. Among caregivers, one study reported improvements in emotional awareness [27], a reported significant increase in parental self-efficacy, expectations for their child’s future, and sense of control over youth outcomes from pre-intervention to 1-month follow-up, despite no significant change in parents’ coping [19].

#### General Mental Health

Three studies reported on general youth MH outcomes: one study found that youth in the intervention group were less likely to receive additional MH diagnosis, be prescribed psychiatric medications, or receive therapy from alternate providers, and were less likely to be involved in criminal justice compared to controls [28]. Another study revealed cultural differences in MH perceptions, with Bhutanese families placing greater emphasis on youth MH improvements, while Somali Bantu families had less discussion of MH impacts [10]. Another study reported a small negative effect size for youth misbehavior [20], suggesting a slight improvement in this area.

#### Youth Substance Use Outcomes

Two studies in this review examined the impact of family-focused interventions on youth SU outcomes, and both reported positive effects in reducing youth SU. The first study evaluated a specialized intervention for youth with comorbid PTSD and SU issues. Authors reported significant reductions in SU days at both 12- and 18-month follow-ups and demonstrated significant decreases in SU, PTSD avoidance, and hyperarousal symptoms compared to the treatment-as-usual group [24]. The second study assessed an intervention specifically designed for LGBTQ youth and their families. Authors reported reduced SU among LGBTQ youth, with improvements in parent support/acceptance, FF, and communication potentially contributing to this reduction [13]. For detailed statistical findings on youth SU outcomes, see Appendix B.

### Family Functioning and/or Parenting Skills

Various outcomes were assessed in appraising FF and/or PS across the 25 studies, with improvements in FF and/or PS reported across multiple areas (see Appendix B).

#### Improved Family Communication and Relationships

Six studies reported significant improvements in family communication and family time together [10, 13, 23, 27, 28, 32]. Four studies reported significant reductions in family conflict and criticism through improved communication [16, 23, 30, 31]. Two studies found that parents gained a better understanding of their youth’s emotions and developed a shared language for discussing feelings, potentially strengthening family bonds and reducing misunderstandings [6, 27]. One study reported increased parent support/acceptance of LGBTQ youth [13]. Another noted that parents reported learning new strategies to navigate challenging parenting situations [6]. One study reported no significant changes in parent–youth relationships [11].

#### Parental Mindfulness

Mixed effects were reported on parental mindfulness: one study reported significant improvements in self-compassion and mindful parenting, with effects maintained at 6-month follow-up [14]. Another study reported improvements in youth perceived social support from family [17]. One study found no significant changes in mindful parenting and child mindfulness levels [11].

#### Parenting Skills

Various programs focused on improving specific PS. Two studies focused on improving parental emotion regulation skills [6, 16]. Two other studies explored specific approaches which resulted in improvements in PS [7, 10]. Other studies reported improvements in transition preparation activities and parental self-efficacy [19], decreases in protective parenting interactions [18], significant reductions in the use of corporal punishment [21], and parents reporting a better understanding of their youths’ emotions [27]. A technology-based intervention demonstrated improvements in various parenting aspects, including increased awareness of FF and self-reported positive behavior changes in decision-making about youth input and parental monitoring [23]. A parent-only group intervention aimed at normalizing parental difficulties and reducing shame reported no specific results [4].

#### Technology-Based Interventions

The integration of technology in family-focused interventions was considered together as a separate category, which yielded mixed results. One study found that a smartphone app for self-monitoring FF led to increased awareness of family dynamics and self-reported positive behavior changes [23]. Another study reported significant reductions in caregiver strain through a technology-enabled pediatric and family behavioral health service [5]. Finally, a study on a hybrid delivery intervention reported no statistically significant changes; authors noted trends in the desired direction [2].

#### Family Dynamics and Support Outcomes

Several studies illuminated specific aspects of family dynamics and/or support. Two studies focused on attachment, with one reporting small negative effect sizes for nurturing and attachment in a post-reunification service [20], while the other reported a significant decrease in caregiver-avoidant attachment but no significant changes in youth–caregiver attachment security [9]. One study each reported improvements in overall family stability and relationship quality [28], youth-perceived familial social support [17], and small positive effect sizes for improvements in social support [20]. Three studies did not report specific outcomes related to family dynamics or support [8, 24, 33]. For detailed statistical findings on family dynamics and support outcomes, see Appendix B.

## Discussion

This scoping review identified 33 strengths-based programs for youth at risk for experiencing toxic stress which targeted youth MH or SU outcomes through improving FF and/or PS described in the literature between 2018 and 2023. Findings highlight the diversity of program characteristics for adversity-impacted youth. The critical findings from this review are summarized in Table 2, highlighting the key discoveries across program characteristics and outcomes.

**Table 2.** Critical Findings.

	Critical Findings of the Review
1	Behavioral frameworks were predominant among identified programs (52%), with emerging evidence supporting multi-framework approaches for youth at risk for toxic stress.
2	Mental health improvements were consistently reported across studies, particularly in youth depression, anxiety, and emotional regulation, while substance use outcomes were understudied (only 12% of programs).
3	Family-focused interventions demonstrated effectiveness through six studies showing improved family communication and four studies reporting significant reductions in family conflict.
4	Demographic representation revealed both progress and gaps: overrepresentation of Black (26%) and Hispanic (26%) participants in U.S. studies, but limited research in low- and middle-income countries and among LGBTQ+ youth.
5	Technology-enhanced interventions showed promise for improving family functioning and mental health outcomes, though findings were mixed and warrant further investigation.

These programs employed various theoretical frameworks, with over half utilizing behavioral approaches. The predominance of behavioral frameworks, particularly DBT and CBT, found among included programs reflects the established efficacy of these frameworks in addressing youth MH challenges within the literature (Jeppesen et al., 2021; Krause et al., 2024; Syversen et al., 2024). However, findings also indicated a trend toward more comprehensive, multi-modal interventions that integrate multiple frameworks. The inclusion of other approaches, including mindfulness, resilience, systemic, social, and developmental approaches, aligns with the growing literature highlighting the complex, multifaceted nature of toxic stress and the need for holistic interventions to mitigate negative sequelae and promote resilience and well-being among adversity-impacted youth (Bethell et al., 2017; Masten & Barnes, 2018). This emphasis on behavioral regulation, while important, may represent a narrow focus given the broad impacts of toxic stress on development. Current literature suggests toxic stress affects multiple domains including physical health, cognitive functioning, and social relationships (McEwen, 2017; Ortiz et al., 2022).

Program duration and frequency varied widely among programs, ranging from single sessions to multi-year interventions. The variability found in program duration and frequency highlights a need for further research to better understand how factors like session number and duration may impact youth outcomes and participant burden. Brief interventions, particularly those that incorporate motivational interviewing, have been particularly efficacious in decreasing adolescent alcohol and SU (Steele et al., 2020). Given the ubiquity of mobile devices, digital micro-interventions provide an additional opportunity to achieve targeted outcomes with youth and their families (Baumel et al., 2020). While the effectiveness of brief, targeted, evidence-based interventions require further exploration within the context of caregiver involvement, the need for prolonged, multi-year interventions may still be necessary due to complex individual, familial, or societal factors. Further research may illuminate whether shorter programs—which may be more feasible and/or cost-effective—may be as impactful as longer interventions, and whether program duration and/or frequency may vary according to case complexity or other considerations.

Of the 31 programs assessing youth and/or caregiver MH outcomes, a majority primarily targeted improvements in youth behavioral regulation while only four programs assessed youth SU outcomes. The most frequently reported positive MH outcomes among these studies included reductions in youth depressive and anxiety symptoms as well as improved youth emotional regulation. Improvements noted in youth MH outcomes align with findings from a recent systematic review, which found family-focused interventions to be effective in improving youth MH outcomes (Wang et al., 2019). Notably, emotional regulation emerged as a key area of improvement among both youth and caregivers. This finding aligns with emerging research highlighting the protective role of self- and co-regulation among adversity-impacted youth and their families (Murray et al, 2019).

Youth SU outcomes also showed improvement among included studies; however, only two studies that targeted youth SU outcomes met the inclusion criteria for this review. This may indicate a critical gap in the literature and a need for more research exploring relational health approaches for mitigating youth SU outcomes, particularly given the increased risk of SU disorders reported among adversity-impacted youth (Carliner et al., 2016; Shin et al., 2018). A recent systematic review illuminated the intricate connections between trauma and SU among Indigenous communities in North America (Spillane et al., 2023). The analysis of multiple studies revealed strong correlations between SU and both historical and contemporary traumatic experiences. Indigenous participants consistently highlighted how intergenerational trauma and cultural disconnection significantly influence SU patterns. The research emphasizes the critical need for culturally responsive interventions that prioritize healing and cultural reconnection as key strategies for addressing SU disorders (Spillane et al., 2023). The limited attention to these broader outcomes, particularly SU despite its known association with early adversity, represents a significant gap between current programming and our understanding of toxic stress impacts. This gap suggests opportunities for expanding outcome measurement in future interventions to better capture the full range of toxic stress effects and intervention benefits.

Approximately one-third of programs focused on improving family relationships and approximately one-third focused on enhancing positive PS. Positive improvements in family dynamics were also found across studies, including improved FF, communication, and/or PS. The most frequently reported positive FF and/or PS outcomes included improvements in family communication, time spent together, and reductions in family conflict. These findings align with a growing research focus on the role of family-based interventions in promoting healthy FF and resilience among adversity-impacted youth (Van Ryzin et al., 2016; Sanders & Turner, 2018) and underscore relational health as a key component among effective interventions for youth at risk for toxic stress. Relational health interventions focused on strengthening FF and enhancing PS may buffer against the negative impacts of toxic stress and may build resilience and promote positive developmental outcomes despite exposure to adversity (Cohen, 2021; Shonkoff et al., 2021).

Caregiver involvement varied similarly, ranging from combined youth–caregiver sessions to separate caregiver-only components, with some programs emphasizing caregiver education and skill-building, while others focused on improving family communication and relationships. Despite varied levels of caregiver involvement found in this review, caregivers play an important role in decreasing symptoms in youth following a traumatic event (Szota et al., 2023). In fact, how parents are involved in interventions may impact intervention outcomes (Dardas et al., 2018). Differences found between caregiver involvement and approaches to caregiver engagement among included programs highlight an opportunity for further research to expand upon differential effects of caregiver involvement and/or engagement approaches among relational health interventions, and how these may ultimately impact youth outcomes.

### Diversity and Representation

Diversity and representation in research are important considerations when developing effective and culturally responsive interventions that address the needs of diverse populations (Bernal & Adames, 2017). The demographic characteristics of participants across included studies highlight important trends in representation that may impact program effectiveness and generalizability. The relatively wide geographical distribution of included programs may indicate a global recognition of the potential of relational health approaches among adversity-impacted youth, as well as a potential for cross-cultural adaptation of these approaches. That most programs were found to be implemented in the United States, Europe, Australia, and Canada highlights a current gap in the literature regarding program availability and implementation across low- and middle-income countries (LMICs), particularly within Africa, South Asia, and the Middle East (Pedersen et al., 2019). Moreover, it is crucial to recognize that the conceptualization of adversity and trauma is not universal but deeply rooted in cultural, social, and historical contexts (Stamm & Friedman, 2000). What constitutes a traumatic experience or ACE may vary significantly across different cultural landscapes. For instance, in Indigenous communities, traumatic experiences are often understood collectively rather than individually. For example, research by Brave Heart and DeBruyn (1998) demonstrated how historical trauma related to colonization and cultural disruption can be transmitted intergenerationally, fundamentally differing from Western psychological frameworks of individual trauma. Western psychological frameworks might not fully capture the complex ways in which communities in Africa, South Asia, and the Middle East understand, experience, and respond to adversity. Indigenous knowledge systems, community support structures, and traditional healing practices may offer alternative perspectives on resilience and coping that are not adequately represented in current intervention models or studied as interventions because they are viewed as a way of life. Future research must adopt a more culturally humble approach, engaging directly with local communities to understand their unique interpretations of adversity, stress, and healing, rather than assuming a one-size-fits-all definition.

Black participants were found to be overrepresented (26%) among included studies relative to the U.S. general population (13.7%; U.S. Census Bureau, 2023). On the surface, this would seem a positive finding given the historical underrepresentation of this group within youth intervention research. Similarly, Hispanic participants were found to be slightly higher in representation (26%) compared to the general U.S. population (19.5%). These findings are notable, as both groups have been found to face unique challenges related to MH, family dynamics, and access to interventions (Bernard et al., 2021; Cleary et al., 2018; Opara et al., 2021). The increased representation of Black and Hispanic youth may reflect a growing effort to include these populations in family-based intervention studies and work toward mitigating long-standing disparities in research participation. More likely, however, these groups are overrepresented in our findings because they are disproportionately exposed to adversity and toxic stress due to systemic factors like poverty, racial discrimination, neighborhood violence, and inadequate resource and social support systems (Beech et al., 2021). Non-Hispanic, White participants were found to be underrepresented among included studies (36% vs. 58.4% in the general U.S. population), and several studies failed to report race or ethnicity or provided ambiguous reports. The gender distribution of participants among included studies (62.6% female, 36.8% male, and 0.2% other) was found to diverge from the general U.S. population’s roughly equal distribution. The inclusion of participants identifying as other genders, while small (0.2%), may represent a step toward inclusivity in research participation. Transgender and gender-diverse youth have been found to be at higher risk for exposure to toxic stress, yet they remain underrepresented in the literature (Newcomb et al., 2020). Only one included program—the Familias con Orgullo (Families with Pride) Intervention, which focused on Latinx sexual minority youth—was found to specifically include LGBTQ youth. This finding underscores a need for further research focused on the needs of adversity-impacted LGBTQ+ youth.

### Methodology and Measurement

Among the 25 research studies included in this review, most employed non-experimental designs, reflecting common methodological challenges in studying complex interventions with vulnerable populations. While this pattern limits our ability to draw strong causal conclusions about program effectiveness, it likely represents practical and ethical considerations in conducting research with adversity-impacted youth rather than the limitations of the programs themselves. To strengthen the evidence base, future research would benefit from incorporating additional methodologies, particularly RCTs with large, diverse samples may help to establish the generalizability of findings (Ungar & Theron, 2020) and longitudinal studies may help to determine the long-term impacts and sustainability of these interventions.

The variability found in both outcome measures and theoretical framework among included studies makes comparison of study findings challenging. This variability reflects a classic challenge in synthesizing intervention research, as different theoretical orientations often lead to different treatment goals and corresponding measurement approaches (Moreau & Wiebels, 2021). The diverse theoretical frameworks employed across studies resulted in varying conceptualizations of success and different approaches to measuring outcomes. While our review found the Perceived Stress Scale and the Difficulties in Emotion Regulation Scale to be the most commonly reported outcome measures, these instruments, though demonstrated good validity and reliability in various contexts (Kaufman et al., 2016; Lee, 2012; Perasso & Velotti, 2017), may present unique considerations when applied to youth experiencing toxic stress. The measurement challenges identified in our review are reinforced by recent research examining toxic stress assessment methods (Parada & Parada, 2024). A comprehensive systematic review documented significant heterogeneity in measurement approaches, identifying over 30 distinct tools and methods currently used to assess toxic stress in young populations (Parada & Parada, 2024). Their analysis revealed critical gaps in measurement standardization and highlighted how this lack of consistency affects both the definition and assessment of toxic stress. The parallel findings between their systematic examination of measurement tools and our review of interventions emphasize the pressing need within the field to develop and validate standardized assessment approaches for evaluating toxic stress in youth populations. Future research should aim to establish a core set of validated, culturally responsive measures to facilitate comparison across studies and populations. This standardization may also enhance the field’s ability to draw more robust conclusions about the efficacy of different intervention approaches.

### Implications for Research, Practice, and Policy

Findings from this review have important implications for research, clinical practice, and policy, though these must be understood within the complex landscape of cultural diversity and resource variability across implementation contexts (See Table 3). Different cultural sub-groups and communities may require distinctly different approaches, and what works in one context may not translate to another (Jagosh, 2019). For example, programs implemented in well-resourced settings (e.g., Swedish communities) face fundamentally different implementation challenges compared to those in under-resourced settings, influenced by factors such as healthcare infrastructure, social support systems, and cultural norms around help-seeking behaviors.

**Table 3.** Implications for Research, Practice, and Policy.

	Implications
Research	- Prioritize rigorous methodologies (e.g., RCTs, longitudinal studies) with diverse samples - Establish a core set of validated, culturally responsive measures - Identify optimal level and type of caregiver involvement for promoting positive outcomes - Assess interventions within the broader context of community and societal factors - Expand research on substance use prevention and intervention approaches - Consider implementation challenges in different contextual environments
Practice	- Incorporate evidence-based relational health approaches into treatment interventions - Prioritize cultural responsiveness and adapting interventions for diverse families - Create safe, supportive, and inclusive environments for families - Adopt a multisystemic approach addressing various factors contributing to toxic stress - Engage and empower caregivers in the intervention process
Policy	- Prioritize funding for research and programs adopting a relational health framework - Support development, evaluation, and scale-up of culturally responsive interventions - Integrate interventions into existing systems of care (schools, clinics, and community organizations) - Address social determinants of health contributing to toxic stress - Reduce disparities in access to high-quality mental health and substance use services - Engage advocates and community stakeholders to promote evidence-based programs and policies - Consider long-term economic and societal benefits of investing in relational health interventions

Findings also have implications for clinical practice. MH providers may consider incorporating evidence-based family interventions, such as those employing cognitive-behavioral therapy, mindfulness, and emotion regulation techniques, into their practice (Sanders & Turner, 2018). When implementing these interventions, it is important for providers to consider cultural responsiveness and adapt these approaches to meet the unique needs and preferences of diverse families (Masten & Barnes, 2018). Implementation efforts may include centering cultural humility, providing language-appropriate services, and addressing barriers to access and engagement (Bounds et al., 2023; Ungar & Theron, 2020).

While the findings of the reviewed studies do not directly emphasize environmental safety as a primary outcome, our analysis suggests broader implications for intervention design. This emphasis on creating safe, supportive, and inclusive environments that foster trust, respect, and open communication with families (Bounds et al., 2023) critically highlights the importance of caregiver engagement and empowerment in promoting positive outcomes among adversity-impacted youth. Researchers and practitioners should aim to incorporate caregivers in interventions and to provide caregivers with the skills, knowledge, and support necessary for them to effectively support youth at risk for toxic stress (Sanders & Turner, 2018). Future research may aim to identify the optimal level and/or type of caregiver involvement needed to promote positive youth and family outcomes.

Findings also support policy initiatives that prioritize the dissemination and implementation of effective family-based interventions that employ a relational health framework and address the complex needs of adversity-impacted youth and their families (Garner et al., 2021). This includes supporting the development, evaluation, and scale-up of culturally responsive interventions, as well as initiatives to reduce disparities in access to high-quality MH and SU services. Policy efforts should center multisystemic approaches that address the various factors contributing to adversity and social determinants of health (Ungar & Theron, 2020) and aim to integrate interventions into existing systems of care (e.g., schools, clinics, and community organizations) (Masten & Barnes, 2018; Van Der Put et al., 2018).

### Strengths, Limitations, and Future Directions

This scoping review has several strengths that enhance the reliability of findings, including a comprehensive literature search strategy, stepwise application of inclusion and exclusion criteria, focus on recently published articles, and data extraction conducted independently by two reviewers with conflicts resolved by the senior author. However, limitations should also be considered when interpreting results. Relevant studies may have been missed due to the selected keywords, subject headings, databases, or other exclusion criteria such as language and publication date range.

A significant limitation of this review is that studies were not required to explicitly identify or measure toxic stress in the methodology. Assumptions were made about the presence of toxic stress risk based on reported trauma exposure, chronic adversity, or related symptoms. While this allowed for the capture of relevant interventions, it also means that some included studies may not have specifically targeted toxic stress mechanisms. Future reviews might consider stricter criteria for identifying studies that explicitly address toxic stress or including only studies that measure biological markers of stress system activation.

Another significant limitation is the inclusion of studies from dramatically different cultural and contextual environments without systematic analysis of how these differences might impact program effectiveness. Programs implemented in well-resourced settings (e.g., affluent U.S. or Swedish communities) may face very different challenges compared to those in under-resourced settings, and their effectiveness may not translate across these contexts (Fredrick, 2018; Sharkey, 2018). We acknowledge our positionality as a diverse group of researchers, many of which come from minoritized, marginalized, and/or under resourced environments, which might have influenced our decision to be inclusive of varied cultural and contextual environments. Future reviews should consider more systematic ways to analyze and compare programs within similar resource contexts or explicitly examine how resource differences impact implementation and outcomes. We do, however, encourage the continued inclusion of studies from lower-resourced environments in systematic reviews given those populations are often disproportionately exposed to adversity and toxic stress.

Another important limitation was the exclusion of dissertations, theses, and non-published program reports from our review. This decision may have introduced publication bias and overlooked valuable evidence from community-based programs that often lack resources for academic publication but may be conducting effective family support work (Al-Ubaydli et al., 2017). Community agencies frequently implement and evaluate family support programs but may not have the capacity or resources to publish their findings in peer-reviewed journals. This limitation reflects a broader challenge in translational science—the gap between academic research and community practice—and may have resulted in missing important insights from real-world program implementation. Additionally, while a scoping review methodology provides a broad overview of the available literature, it does not provide an in-depth synthesis or quality appraisal of included studies.

Future research directions may address these limitations and build upon findings from this review. Future research should move beyond simply calling for larger samples and experimental research designs to focus on understanding how programs work in specific cultural and contextual environments. This includes examining researcher positionality and its impact on program development and evaluation. While RCTs with diverse samples can help establish program efficacy (Ungar & Theron, 2020), researchers must also investigate how cultural variables influence outcomes and document not just if programs work, but for whom they work, under what conditions, and why (Jagosh, 2019). The development and evaluation of culturally responsive and contextually relevant interventions should also be prioritized, along with the identification of cultural factors that may impact program effectiveness and engagement. Qualitative research and participatory methodologies may help to illuminate the unique experiences and/or perspectives of adversity-impacted youth and families. Findings support further research regarding the potential of technology-based interventions to increase access and/or engagement among adversity-exposed youth and families, as well as establishing a core set of validated, culturally responsive measures to facilitate comparisons across studies and populations. Future research efforts should aim to bridge the gap between academic research and community practice by including evidence from non-academic sources such as program evaluation reports, dissertations, and community agency documentation. This could provide valuable insights into real-world implementation challenges and successes, particularly among community-based organizations serving diverse populations. Establishing partnerships between academic researchers and community agencies could help facilitate the documentation and dissemination of practice-based evidence (Clark et al., 2023). Future studies may also examine relationships between session number, duration, and long-term outcomes. Critically, more research is needed on relational health-based programs that target youth SU prevention, as well as research in LMICs to support youth MH needs and develop culturally-responsive and contextually relevant interventions. Future research may also aim to explore the effectiveness of single versus multi-framework approaches, how different theoretical orientations might best match specific youth populations and/or types of toxic stress experiences, and to identify the optimal level and/or type of caregiver involvement necessary to promote positive youth and family outcomes.

## Conclusion

This scoping review highlights the positive potential of strength-based, relational health interventions in promoting youth and caregiver MH, preventing youth SU, and improving FF and/or PS among adversity-impacted youth and their families. Findings support further development, evaluation, and dissemination of relational health-based interventions to meet the diverse needs of adversity-impacted youth and their families, with particular attention to addressing systemic inequities and ensuring accessibility across different communities and contexts.

## Supplemental Material

sj-docx-1-tva-10.1177_15248380251326902 – Supplemental material for Strengths-Based Programs for Youth at Risk for Toxic Stress: A Scoping Review of Programs Targeting Mental Health, Substance Use, Parenting Skills, and Family Functioning Supplemental material, sj-docx-1-tva-10.1177_15248380251326902 for Strengths-Based Programs for Youth at Risk for Toxic Stress: A Scoping Review of Programs Targeting Mental Health, Substance Use, Parenting Skills, and Family Functioning by Afsaneh Saghafi, Sarah M. Rodrigues, Jayla Aldridge, Maruko Myint, Donna Balsam, Nayeli Inzunza, Julissa Hernandez, Stephen L. Clancy, Luis Monreal-Duarte and Dawn T. Bounds in Trauma, Violence, & Abuse

## References

[bibr1-15248380251326902] Al-Ubaydli O. List J. A. Suskind D. L. (2017). What can we learn from experiments? Understanding the threats to the scalability of experimental results. American Economic Review, 107(5), 282–286. 10.1257/aer.p20171115

[bibr2-15248380251326902] Arksey H. O’Malley L. (2005). Scoping studies: Towards a methodological framework. International Journal of Social Research Methodology, 8(1), 19–32. 10.1080/1364557032000119616

[bibr3-15248380251326902] *Arnaud N. Baldus C. Laurenz L. J. Bröning S. Brandt M. Kunze S. Austermann M. Zimmermann L. Daubmann A. Thomasius R. (2020). Does a mindfulness-augmented version of the German Strengthening Families Program reduce substance use in adolescents? Study protocol for a randomized controlled trial. Trials, 21(1), 114. 10.1186/s13063-020-4065-1 31992356 PMC6988370

[bibr4-15248380251326902] Banks D. E. Brown K. Saraiya T. C. (2023). “Culturally responsive” substance use treatment: contemporary definitions and approaches for minoritized racial/ethnic groups. Current Addiction Reports, 10(3), 422–431. 10.1007/s40429-023-00489-0 41602173 PMC12834561

[bibr5-15248380251326902] Baumel A. Fleming T. Schueller S. M. (2020). Digital micro interventions for behavioral and mental health gains: core components and conceptualization of digital micro intervention care. Journal of Medical Internet Research, 22(10), e20631. 10.2196/20631 PMC7661243 33118946

[bibr6-15248380251326902] Beech B. M. Ford C. Thorpe R. J. Bruce M. A. Norris K. C. (2021). Poverty, racism, and the public health crisis in America. Frontiers in Public Health, 9, 699049. 10.3389/fpubh.2021.699049 34552904 PMC8450438

[bibr7-15248380251326902] Bernal G. Adames C. (2017). Cultural adaptations: conceptual, ethical, contextual, and methodological issues for working with ethnocultural and majority-world populations. Prevention Science, 18(6), 681–688. 10.1007/s11121-017-0806-0 28573426

[bibr8-15248380251326902] Bernard D. L. Calhoun C. D. Banks D. E. Halliday C. A. Hughes-Halbert C. Danielson C. K. (2021). Making the “C-ACE” for a Culturally-Informed Adverse Childhood Experiences Framework to Understand the Pervasive Mental Health Impact of Racism on Black Youth. Journal of Child & Adolescent Trauma, 14(2), 233–247. 10.1007/s40653-020-00319-9 33986909 PMC8099967

[bibr9-15248380251326902] *Besse-Flütsch N. Bühlmann C. Fabijani N. Ruschetti G. G. Smigielski L. Pauli D. (2023). Home treatment as an add-on to family-based treatment for adolescents with anorexia nervosa compared with standard family-based treatment and home-based stress reduction training: Study protocol for a randomized clinical trial. Journal of Eating Disorders, 11(1), 135. 10.1186/s40337-023-00861-5 37580810 PMC10424408

[bibr10-15248380251326902] Bethell C. D. Solloway M. R. Guinosso S. Hassink S. Srivastav A. Ford D. Simpson L. A. (2017). Prioritizing possibilities for child and family health: an agenda to address adverse childhood experiences and foster the social and emotional roots of well-being in pediatrics. Academic Pediatrics, 17(7), S36–S50. 10.1016/j.acap.2017.06.002 28865659

[bibr11-15248380251326902] Bomysoad R. N. Francis L. A. (2020). Adverse childhood experiences and mental health conditions among adolescents. Journal of Adolescent Health, 67(6), 868–870. 10.1016/j.jadohealth.2020.04.013 32576484

[bibr12-15248380251326902] Bounds D. T. Posey P. D. (2022). A resistance framework for racially minoritized youth behaviors during the transition to adulthood. Journal of Research on Adolescence, 32(3), 959–980. 10.1111/jora.12792 35980807 PMC9543550

[bibr13-15248380251326902] Bounds D. T. Rodrigues S. M. Milburn N. G. (2023). Strengthening families to disrupt intergenerational health inequities with adolescents at risk for commercial sexual exploitation, substance use, and HIV. American Journal of Public Health, 113(S2), S124–S128. 10.2105/AJPH.2023.307284 PMC10282847 37339412

[bibr14-15248380251326902] Bounds D. T. Winiarski D. A. Otwell C. H. Tobin V. Glover A. C. Melendez A. Karnik N. S. (2020). Considerations for working with youth with socially complex needs. Journal of Child and Adolescent Psychiatric Nursing, 33(4), 209–220. 10.1111/jcap.12288 32691491 PMC7970826

[bibr15-15248380251326902] Brave Heart M. Y. DeBruyn L. M . (1998). The American Indian Holocaust: Healing historical unresolved grief. American Indian and Alaska Native Mental Health Research: Journal of the National Center, 8(2), 60–82. 10.5820/aian.0802.1998.60 9842066

[bibr16-15248380251326902] *Breaux R. Shroff D. M. Cash A. R. Swanson C. S. Carlton C. Bertollo J. R. Dahiya A. V. (2023). Telehealth delivery of the RELAX intervention for families of adolescents diagnosed with ADHD: Preliminary treatment outcomes and evidence of acceptability and feasibility. Evidence-Based Practice in Child and Adolescent Mental Health, 8(1), 24–38. 10.1080/23794925.2021.1970053

[bibr17-15248380251326902] *Brown F. L. Bosqui T. Elias J. Farah S. Mayya A. Abo Nakkoul D. Walsh B. Chreif S. Einein A. Meksassi B. Abi Saad R. Naal H. Ghossainy M. E. Donnelly M. Betancourt T. S. Carr A. Puffer E. El Chammay R. Jordans M. J. D. (2022). Family systemic psychosocial support for at-risk adolescents in Lebanon: Study protocol for a multi-site randomized controlled trial. Trials, 23(1), 327. 10.1186/s13063-022-06284-y 35436976 PMC9014280

[bibr18-15248380251326902] Carliner H. Keyes K. M. McLaughlin K. A. Meyers J. L. Dunn E. C. Martins S. S. (2016). Childhood trauma and illicit drug use in adolescence: A population-based national comorbidity survey replication–adolescent supplement study. Journal of the American Academy of Child & Adolescent Psychiatry, 55(8), 701–708. 10.1016/j.jaac.2016.05.010 27453084 PMC4964281

[bibr19-15248380251326902] Center for Disease Control and Prevention. (2021). Adverse childhood experiences prevention strategy. National Center for Injury Prevention and Control, Centers for Disease Control and Prevention. https://www.cdc.gov/aces/prevention/index.html

[bibr20-15248380251326902] Clark R. Gaber J. Datta J. Talat S. Bomze S. Marentette-Brown S. Gagnon C. Oliver D. Lamarche L. Forsyth P. Carr T. Price D. Mangin D. (2023). Understanding collaborative implementation between community and academic partners in a complex intervention: A qualitative descriptive study. BMC Health Services Research, 23(1), 606. 10.1186/s12913-023-09617-y 37296452 PMC10257302

[bibr21-15248380251326902] *Clauss J. A. Bhiku K. Burke A. Pimentel-Diaz Y. DeTore N. R. Zapetis S. Zvonar V. Kritikos K. Canenguez K. M. Cather C. Holt D. J. (2023). Development of a transdiagnostic, resilience-focused intervention for at-risk adolescents. Journal of Mental Health, 32(3), 592–601. 10.1080/09638237.2022.2140790 36369940 PMC10175511

[bibr22-15248380251326902] Cleary S. D. Snead R. Dietz-Chavez D. Rivera I. Edberg M. C. (2018). Immigrant Trauma and Mental Health Outcomes Among Latino Youth. Journal of Immigrant and Minority Health, 20(5), 1053–1059. 10.1007/s10903-017-0673-6 29139024 PMC6436088

[bibr23-15248380251326902] *Cloutier P. Gray C. Sheridan N. Silverman A. Cappelli M. Zemek R. Jabbour M. Reid S. Kennedy A. (2022). Building Resilience and Attachment in Vulnerable Adolescents (BRAVA): A brief group intervention for adolescents with mild-to-moderate suicidal ideation and their caregivers. Child and Adolescent Mental Health, 27(4), 343–351. 10.1111/CAMH.12506 34498386

[bibr24-15248380251326902] Cohen. (2021, April). Three principles to improve outcomes for children and familie. Center on the Developing Child at Harvard University. http://www.developingchild.harvard.edu/

[bibr25-15248380251326902] *Colegrove V. M. Havighurst S. S. Kehoe C. E. (2019). Emotion regulation during conflict interaction after a systemic music intervention: Understanding changes for parents with a trauma history and their adolescent. Nordic Journal of Music Therapy, 28(5), 405–425. 10.1080/08098131.2019.1616807

[bibr26-15248380251326902] Covidence systematic review software. (2024). [Computer software]. Veritas Health Innovation, Melbourne, Australia. www.covidence.org

[bibr27-15248380251326902] *Danielson C. K. Adams Z. McCart M. R. Chapman J. E. Sheidow A. J. Walker J. Smalling A. De Arellano M. A. (2020). Safety and efficacy of exposure-based risk reduction through family therapy for co-occurring substance use problems and posttraumatic stress disorder symptoms among adolescents: A randomized clinical trial. JAMA Psychiatry, 77(6), 574. 10.1001/jamapsychiatry.2019.4803 32022827 PMC7042939

[bibr28-15248380251326902] Dardas L. A. van de Water B. Simmons L. A. (2018). Parental involvement in adolescent depression interventions: A systematic review of randomized clinical trials. International Journal of Mental Health Nursing, 27(2), 555–570. 10.1111/inm.12429 29277947

[bibr29-15248380251326902] Duke N. N. (2018). Adolescent adversity and concurrent tobacco, alcohol, and marijuana use. American Journal of Health Behavior, 42(5), 85–99. 10.5993/AJHB.42.5.8 30688644

[bibr30-15248380251326902] Felitti V. J. Anda R. F. Nordenberg D. Williamson D. F. Spitz A. M. Edwards V. Koss M. P. Marks J. S. (1998). Relationship of childhood abuse and household dysfunction to many of the leading causes of death in adults. The Adverse Childhood Experiences (ACE) Study. American Journal of Preventive Medicine, 14(4), 245–258. 10.1016/s0749-3797(98)00017-8 9635069

[bibr31-15248380251326902] Fredrick E. (2018). Death, violence, health, and poverty in Chicago. Harvard Public Health Review, 19, 1–25. 10.54111/0001/S1

[bibr32-15248380251326902] Garbarino J. Kostelny K. Dubrow N. (1991). What children can tell us about living in danger. American Psychologist, 46(4), 376–383. 10.1037/0003-066X.46.4.376 2048796

[bibr33-15248380251326902] Garner A. Yogman M. , & Committee on Psychosocial Aspects of Child and Family Health, S. O. D. A. B. P., Council on Early Childhood. (2021). Preventing childhood toxic stress: partnering with families and communities to promote relational health. Pediatricss 148(2), e2021052582. 10.1542/peds.2021-052582 34312296

[bibr34-15248380251326902] *Gill D. Warburton W. Simes D. Sweller N. (2018). Group therapy for emotional dysregulation: Treatment for adolescents and their parents. Child and Adolescent Social Work Journal, 35(2), 169–180. 10.1007/S10560-017-0510-8

[bibr35-15248380251326902] *Haar K. El-Khani A. Lodi R. Molin V. Pelosi A. Yassine A. Campello G. Maalouf W. (2023). Assessing the efficacy of a brief universal family skills programme on violence and substance-use indicators in youth in Trentino and Parma, Italy: Study protocol for a multi-centre, non-blinded, cluster-randomised controlled trial (cRCT) of family united. International Journal of Environmental Research and Public Health, 20(16), 6548. 10.3390/ijerph20166548 PMC10454720 37623134

[bibr36-15248380251326902] Hoffmann J. P. Jones M. S. (2022). Cumulative stressors and adolescent substance use: A review of 21st-century literature. Trauma, Violence, & Abuse, 23(3), 891–905. 10.1177/1524838020979674 33345723

[bibr37-15248380251326902] *Holmqvist Larsson K. Lowén A. Hellerstedt L. Bergcrona L. Salerud M. Zetterqvist M . (2020). Emotion regulation group skills training: A pilot study of an add-on treatment for eating disorders in a clinical setting. Journal of Eating Disorders, 8(1). 10.1186/S40337-020-00289-1 PMC7118896 32266070

[bibr38-15248380251326902] Hughes K. Bellis M. A. Hardcastle K. A. Sethi D. Butchart A. Mikton C. Jones L. Dunne M. P. (2017). The effect of multiple adverse childhood experiences on health: A systematic review and meta-analysis. The Lancet. Public Health, 2(8), e356–e366. 10.1016/S2468-2667(17)30118-4 29253477

[bibr39-15248380251326902] Jagosh J. (2019). Realist synthesis for public health: building an ontologically deep understanding of how programs work, for whom, and in which contexts. Annual Review of Public Health, 40, 361–372. 10.1146/annurev-publhealth-031816-044451 30633712

[bibr40-15248380251326902] Jeppesen P. Wolf R. T. Nielsen S. M. Christensen R. Plessen K. J. Bilenberg N. Thomsen P. H. Thastum M. Neumer S.-P. Puggaard L. B. Agner Pedersen M. M. Pagsberg A. K. Silverman W. K. Correll C. U. (2021). Effectiveness of transdiagnostic cognitive-behavioral psychotherapy compared with management as usual for youth with common mental health problems: A randomized clinical trial. JAMA Psychiatry, 78(3), 250–260. 10.1001/jamapsychiatry.2020.4045 33355633 PMC7758821

[bibr41-15248380251326902] *Kaufman E. A. Clerke A. S. Meddaoui B. (2023). Translating core intervention strategies into action: Interpersonal validation among self-injuring adolescents and their mothers. Journal of Clinical Psychology, 79(1), 105–125. 10.1002/JCLP.23393 35611597

[bibr42-15248380251326902] Kaufman E. A. Xia M. Fosco G. Yaptangco M. Skidmore C. R. Crowell S. E. (2016). The Difficulties in Emotion Regulation Scale Short Form (DERS-SF): Validation and Replication in Adolescent and Adult Samples. Journal of Psychopathology and Behavioral Assessment, 38(3), 443–455. 10.1007/s10862-015-9529-3 41522882 PMC12788809

[bibr43-15248380251326902] *Kirby A. V. Feldman K. J. C. Himle M. B. Diener M. L. Wright C. A. Hoffman J. M. (2021). Pilot test of the maximizing adolescent post-secondary success (MAPSS) intervention: supporting parents of autistic youth. The American Journal of Occupational Therapy, 75(3). 10.5014/AJOT.2021.045815 PMC8095705 34781348

[bibr44-15248380251326902] *Knight D. K. Yang Y. Joseph E. D. Tinius E. Young S. Shelley L. T. Cross D. R. Knight K. (2021). Preventing opioid use among justice-involved youth as they transition to adulthood: Leveraging safe adults (LeSA). BMC Public Health, 21(1), 2133. 10.1186/s12889-021-12127-3 34801009 PMC8605598

[bibr45-15248380251326902] Koita K. Long D. Hessler D. Benson M. Daley K. Bucci M. Thakur N. Burke Harris N. (2018). Development and implementation of a pediatric adverse childhood experiences (ACEs) and other determinants of health questionnaire in the pediatric medical home: A pilot study. PLoS ONE, 13(12), e0208088. 10.1371/journal.pone.0208088 PMC6291095 30540843

[bibr46-15248380251326902] Krause K. Zhang X. C. Schneider S. (2024). Long-term effectiveness of cognitive behavioral therapy in routine outpatient care for youth with anxiety disorders. Psychotherapy and Psychosomatics, 93(3), 181–190. 10.1159/000537932 38615662 PMC11151973

[bibr47-15248380251326902] *Lavner J. A. Barton A. W. Adesogan O. Beach S. R. H. (2021). Family-centered prevention buffers the effect of financial strain on parenting interactions, reducing youth conduct problems in African American families. Journal of Consulting and Clinical Psychology, 89(9), 783–791. 10.1037/ccp0000680 34591551 PMC8862116

[bibr48-15248380251326902] Lee E.-H. (2012). Review of the psychometric evidence of the perceived stress scale. Asian Nursing Research, 6(4), 121–127. 10.1016/j.anr.2012.08.004 25031113

[bibr49-15248380251326902] *Li M. J. Hardy J. Calanche L. Dominguez K. DiStefano A. S. Black D. S. Unger J. B. Chou C.-P. Baezconde-Garbanati L. Contreras R. Bluthenthal R. N. (2021). Initial efficacy of a community-derived mindfulness-based intervention for Latinx parents and their children. Journal of Immigrant and Minority Health, 23(5), 993–1000. 10.1007/s10903-021-01154-2 33575977 PMC8355252

[bibr50-15248380251326902] *Loo T. Hunt J. Grodberg D. Bravata D. (2023). Clinical and psychosocial outcomes associated with a tele-behavioral health platform for families: retrospective study. JMIR Formative Research, 7, e43600. 10.2196/43600 PMC10131771 36930214

[bibr51-15248380251326902] *Lozano A. Estrada Y. Tapia M. I. Dave D. J. Marquez N. Baudin S. Prado G. (2022). Development of a family-based preventive intervention for Latinx sexual minority youth and their parents. Cultural Diversity & Ethnic Minority Psychology, 28(2), 227–239. 10.1037/CDP0000506 34735168

[bibr52-15248380251326902] *Lu S. Lyu R. Hu H. Ho K. K. M. Barry T. J. Black D. Wong D. F. K. (2022). Parallel parent–child mindfulness intervention among Chinese migrant families: A mixed-methods feasibility study. Research on Social Work Practice, 32(8), 925–939. 10.1177/10497315221089684

[bibr53-15248380251326902] Lukoševičiūtė-Barauskienė J. Žemaitaitytė M. Šūmakarienė V. Šmigelskas K. (2023). Adolescent perception of mental health: it’s not only about oneself, it’s about others too. Children, 10(7), 1109. 10.3390/children10071109 PMC10378269 37508606

[bibr54-15248380251326902] Masten A. S. Barnes A. J. (2018). Resilience in children: developmental perspectives. Children, 5(7), 98. 10.3390/children5070098 30018217 PMC6069421

[bibr55-15248380251326902] *Mayoral M. Valencia F. Calvo A. Roldan L. Espliego A. Rodriguez-Toscano E. Kehrmann L. Arango C. Delgado C. (2020). Development of an early intervention programme for adolescents with emotional dysregulation and their families: Actions for the treatment of adolescent personality (ATraPA). Early Intervention in Psychiatry, 14(5), 619–624. 10.1111/EIP.12934 32026614

[bibr56-15248380251326902] *McCullough E. Mathura A. (2019). A comparison between a Neuro-Physiological Psychotherapy (NPP) treatment group and a control group for children adopted from care: Support for a neurodevelopmentally informed approach to therapeutic intervention with maltreated children. Child Abuse & Neglect, 97, 104128. 10.1016/J.CHIABU.2019.104128 31525563

[bibr57-15248380251326902] McEwen B. S. (2017). Neurobiological and systemic effects of chronic stress. Chronic Stress (Thousand Oaks, Calif.), 1, 2470547017692328. 10.1177/2470547017692328 PMC5573220 28856337

[bibr58-15248380251326902] Murray D. W. Rosanbalm K. Christopoulos C. Meyer A. L. (2019). An applied contextual model for promoting self-regulation enactment across development: implications for prevention, public health and future research. The Journal of Primary Prevention, 40(4), 367–403. 10.1007/s10935-019-00556-1 31372788

[bibr59-15248380251326902] Moreau D. Wiebels K. (2021). Assessing change in intervention research: The benefits of composite outcomes. Advances in Methods and Practices in Psychological Science, 4(1), 2515245920931930. 10.1177/2515245920931930

[bibr60-15248380251326902] Nelson C. A. Bhutta Z. A. Burke Harris N. Danese A. Samara M. (2020). Adversity in childhood is linked to mental and physical health throughout life. BMJ, m3048. 10.1136/bmj.m3048 PMC7592151 33115717

[bibr61-15248380251326902] *Neville S. E. DiClemente-Bosco K. Chamlagai L. K. Bunn M. Freeman J. Berent J. M. Gautam B. Abdi A. Betancourt T. S. (2022). Investigating outcomes of a family strengthening intervention for resettled Somali Bantu and Bhutanese refugees: An explanatory sequential mixed methods study. International Journal of Environmental Research and Public Health, 19(19), 12415. 10.3390/IJERPH191912415 36231735 PMC9566609

[bibr62-15248380251326902] Newcomb M. E. Hill R. Buehler K. Ryan D. T. Whitton S. W. Mustanski B. (2020). High burden of mental health problems, substance use, violence, and related psychosocial factors in transgender, non-binary, and gender diverse youth and young adults. Archives of Sexual Behavior, 49(2), 645–659. 10.1007/s10508-019-01533-9 31485801 PMC7018588

[bibr63-15248380251326902] Opara I. Weissinger G. M. Lardier D. T. Lanier Y. Carter S. Brawner B. M. (2021). Mental health burden among Black adolescents: The need for better assessment, diagnosis and treatment engagement. Social Work in Mental Health, 19(2), 88–104. 10.1080/15332985.2021.1879345 34248423 PMC8262091

[bibr64-15248380251326902] Ortiz R. Gilgoff R. Burke Harris N. (2022). Adverse childhood experiences, toxic stress, and trauma-informed neurology. JAMA Neurology, 79(6), 539–540. 10.1001/jamaneurol.2022.0769 35467693

[bibr65-15248380251326902] Parada M. L. Parada J. L. (2024). Measuring toxic stress in childhood and youth: A systematic review. Journal of Pediatric Health Care, 38(6), 836–849. 10.1016/j.pedhc.2024.08.008 39306787

[bibr66-15248380251326902] Parra-Cardona R. Leijten P. Lachman J. M. Mejía A. Baumann A. A. Amador Buenabad N. G. Cluver L. Doubt J. Gardner F. Hutchings J. Ward C. L. Wessels I. M. Calam R. Chavira V. Domenech Rodríguez M. M. (2021). Strengthening a culture of prevention in low- and middle-income countries: Balancing scientific expectations and contextual realities. Prevention Science, 22(1), 7–17. 10.1007/s11121-018-0935-0 30058025 PMC6353698

[bibr67-15248380251326902] Pedersen G. A. Smallegange E. Coetzee A. Hartog K. Turner J. Jordans M. J. D. Brown F. L. (2019). A systematic review of the evidence for family and parenting interventions in low- and middle-income countries: child and youth mental health outcomes. Journal of Child and Family Studies, 28(8), 2036–2055. 10.1007/s10826-019-01399-4

[bibr68-15248380251326902] Perasso G. Velotti P. (2017). Difficulties in emotion regulation scale. In Zeigler-Hill V. Shackelford T. K. (Eds.), Encyclopedia of personality and individual differences (pp. 1–3). Springer International Publishing. 10.1007/978-3-319-28099-8_810-1

[bibr69-15248380251326902] *Ridings L. E. Anton M. T. Winkelmann J. Davidson T. M. Wray L. Streck C. J. Ruggiero K. J. (2019). Trauma resilience and recovery program: addressing mental health in pediatric trauma centers. Journal of Pediatric Psychology, 44(9), 1046–1056. 10.1093/jpepsy/jsz053 31298276 PMC9432141

[bibr70-15248380251326902] *Rushovich B. Sepulveda K. Efetevbia V. Malm K. (2021). A post-reunification service model: Implementation and population served. Children and Youth Services Review, 122, 105928. 10.1016/j.childyouth.2021.105928

[bibr71-15248380251326902] Sanders M. R. Turner K. M. T. (2018). The importance of parenting in influencing the lives of children. In Sanders M. R. Morawska A. (Eds.), Handbook of parenting and child development across the lifespan (pp. 3–26). Springer International Publishing. 10.1007/978-3-319-94598-9_1

[bibr72-15248380251326902] Sharkey P. (2018). Uneasy Peace: The great crime decline, the renewal of city life, and the next war on violence. W.W. Norton; Company. http://www.tinyurl.com/yyvc2qs5

[bibr73-15248380251326902] Shin S. H. McDonald S. E. Conley D. (2018). Patterns of adverse childhood experiences and substance use among young adults: A latent class analysis. Addictive Behaviors, 78, 187–192. 10.1016/j.addbeh.2017.11.020 29179155 PMC5783745

[bibr74-15248380251326902] Shonkoff J. P. Garner A. S. (2012). The lifelong effects of early childhood adversity and toxic stress. Pediatrics, 129(1), e232-246. 10.1542/peds.2011-2663 22201156

[bibr75-15248380251326902] Shonkoff J. P. Slopen N. Williams D. R. (2021). Early childhood adversity, toxic stress, and the impacts of racism on the foundations of health. Annual Review of Public Health, 42, 115–134. 10.1146/annurev-publhealth-090419-101940 33497247

[bibr76-15248380251326902] *Siebelink N. M. Bögels S. M. Speckens A. E. M. Dammers J. T. Wolfers T. Buitelaar J. K. Greven C. U. (2022). A randomized controlled trial (MindChamp) of a mindfulness-based intervention for children with ADHD and their parents. Journal of Child Psychology and Psychiatry, and Allied Disciplines, 63(2), 165–177. 10.1111/JCPP.13430 34030214 PMC9292876

[bibr77-15248380251326902] *Silva D. J. Petrilla C. M. Matteson D. Mannion S. Huggins S. L. (2020). Increasing resilience in youth and families: YAP’s wraparound advocate service model. Child & Youth Services, 41(1), 51–82. 10.1080/0145935X.2019.1610870

[bibr78-15248380251326902] Spillane N. S. Schick M. R. Kirk-Provencher K. T. Nalven T. Goldstein S. C. Crawford M. C. Weiss N. H. (2023). Trauma and substance use among indigenous peoples of the United States and Canada: A scoping review. Trauma, Violence & Abuse, 24(5), 3297–3312. 10.1177/15248380221126184 PMC12109140 36197078

[bibr79-15248380251326902] Stamm B. H. Friedman M. J. (2000). Cultural diversity in the appraisal and expression of trauma. In Shalev A. Y. Yehuda R. McFarlane A. C. (Eds.), International handbook of human response to trauma (pp. 69–85). Springer US. 10.1007/978-1-4615-4177-6_5

[bibr80-15248380251326902] Steele D. W. Becker S. J. Danko K. J. Balk E. M. Adam G. P. Saldanha I. J. Trikalinos T. A. (2020). Brief behavioral interventions for substance use in adolescents: A meta-analysis. Pediatrics, 146(4), e20200351. 10.1542/peds.2020-0351 32928988

[bibr81-15248380251326902] *Stover C. S. Hahn H. Maciejewski K. R. Epstein C. Marans S. (2022). The child and family traumatic stress intervention: Factors associated with symptom reduction for children receiving treatment. Child Abuse & Neglect, 134, 105886. 10.1016/j.chiabu.2022.105886 36152531

[bibr82-15248380251326902] Substance Abuse and Mental Health Services Administration. (2014). SAMHSA’s concept of trauma and guidance for a trauma-informed approach. HHS Publication No. (SMA) 14-4884. Substance Abuse and Mental Health Services Administration. https://store.samhsa.gov/product/samhsas-concept-trauma-and-guidance-trauma-informed-approach/sma14-4884

[bibr83-15248380251326902] Swedo E. A. Sumner S. A. De Fijter S. Werhan L. Norris K. Beauregard J. L. Montgomery M. P. Rose E. B. Hillis S. D. Massetti G. M. (2020). Adolescent opioid misuse attributable to adverse childhood experiences. The Journal of Pediatrics, 224, 102–109.e3. 10.1016/j.jpeds.2020.05.001 PMC8253221 32437756

[bibr84-15248380251326902] *Swendeman D. Sumstine S. Brink A. Mindry D. Medich M. Russell M. (2020). Smartphone self-monitoring by young adolescents and parents to assess and improve family functioning: Qualitative feasibility study. JMIR Formative Research, 4(6), e15777. 10.2196/15777 PMC7381003 32574148

[bibr85-15248380251326902] Syversen A. M. Schønning V. Fjellheim G. S. Elgen I. Wergeland G. J. (2024). Evaluation of dialectical behavior therapy for adolescents in routine clinical practice: A pre-post study. BMC Psychiatry, 24, 447. 10.1186/s12888-024-05876-z 38877441 PMC11177375

[bibr86-15248380251326902] Szota K. Schulte K. L. Christiansen H. (2023). Interventions involving caregivers for children and adolescents following traumatic events: A systematic review and meta-analysis. Clinical Child and Family Psychology Review, 26(1), 17–32. 10.1007/s10567-022-00415-2 36161385 PMC9879828

[bibr87-15248380251326902] *Taliercio J. R. Wigod T. Shen J. Yang L. Davino S. Servidio E. McGinn L. K. Miller A. L. (2023). Coping with transitions: A promising intensive outpatient DBT program for emerging adults and their families. Journal of Contemporary Psychotherapy, 53(4), 349–357. 10.1007/s10879-023-09583-w PMC10193327 37363718

[bibr88-15248380251326902] Thakur N. Hessler D. Koita K. Ye M. Benson M. Gilgoff R. Bucci M. Long D. Burke Harris N. (2020). Pediatrics adverse childhood experiences and related life events screener (PEARLS) and health in a safety-net practice. Child Abuse & Neglect, 108, 104685. 10.1016/j.chiabu.2020.104685 32898839 PMC9350954

[bibr89-15248380251326902] *Thulin J. Nilsson D. Svedin C. G. Kjellgren C. (2020). Outcomes of CPC-CBT in Sweden concerning psychosocial well-being and parenting practice: Children’s perspectives. Research on Social Work Practice, 30(1), 65–73. 10.1177/1049731519843352

[bibr90-15248380251326902] Tricco A. C. Lillie E. Zarin W. O’Brien K. K. Colquhoun H. Levac D. Moher D. Peters M. D. J. Horsley T. Weeks L. Hempel S. Akl E. A. Chang C. McGowan J. Stewart L. Hartling L. Aldcroft A. Wilson M. G. Garritty C. , . . . Straus S. E. (2018). PRISMA extension for scoping reviews (PRISMA-ScR): Checklist and explanation. Annals of Internal Medicine, 169(7), 467–473. 10.7326/M18-0850 30178033

[bibr91-15248380251326902] Ungar M. (2006). Nurturing hidden resilience in at-risk youth in different cultures. Journal of the Canadian Academy of Child and Adolescent Psychiatry, 15(2), 53–58. 18392194 PMC2277285

[bibr92-15248380251326902] Ungar M. Theron L. (2020). Resilience and mental health: How multisystemic processes contribute to positive outcomes. The Lancet Psychiatry, 7(5), 441–448. 10.1016/S2215-0366(19)30434-1 31806473

[bibr93-15248380251326902] U.S. Census Bureau. (2023). QuickFacts: United States. Retrieved April 15, 2024, from https://www.census.gov/quickfacts/fact/table/US/PST045222

[bibr94-15248380251326902] Van Der Put C. E. Assink M. Gubbels J. Boekhout Van Solinge N. F . (2018). Identifying effective components of child maltreatment interventions: A meta-analysis. Clinical Child and Family Psychology Review, 21(2), 171–202. 10.1007/s10567-017-0250-5 29204796 PMC5899109

[bibr95-15248380251326902] *Van Der Westhuizen D. Claassen N. Viljoen M . (2018). A case study of two adolescent–parent pairs describing the association between vagal tone and social-emotional adjustment during a Positive Cognitive Behaviour Therapy Programme. Journal of Child & Adolescent Mental Health, 30(2), 111–130. 10.2989/17280583.2018.1488718 30236037

[bibr96-15248380251326902] Van Ryzin M. J. Roseth C. J. Fosco G. M. Lee Y. Chen I.-C . (2016). A component-centered meta-analysis of family-based prevention programs for adolescent substance use. Clinical Psychology Review, 45, 72–80. 10.1016/j.cpr.2016.03.007 27064553 PMC4856569

[bibr97-15248380251326902] Wang J. Z. Mott S. Magwood O. Mathew C. Mclellan A. Kpade V. Gaba P. Kozloff N. Pottie K. Andermann A. (2019). The impact of interventions for youth experiencing homelessness on housing, mental health, substance use, and family cohesion: A systematic review. BMC Public Health, 19(1), 1528. 10.1186/s12889-019-7856-0 31727031 PMC6857126

[bibr98-15248380251326902] Werner E. E. Smith R. S. (1982). Vulnerable, but invincible: A longitudinal study of resilient children and youth. McGraw-Hill.

[bibr99-15248380251326902] *Wijana M. B. Enebrink P. Liljedahl S. I. Ghaderi A. (2018). Preliminary evaluation of an intensive integrated individual and family therapy model for self-harming adolescents. BMC Psychiatry, 18(1), 371. 10.1186/s12888-018-1947-9 30477463 PMC6258142

[bibr100-15248380251326902] *Wijesekera K. Kiff C. Aralis H. Sinclair M. Bursch B. Alejos J. C. Lester P. (2023). Hybrid delivery of behavioral health screening and prevention intervention for pediatric heart transplant recipients and families: A randomized pilot study. Pediatric Transplantation, 27(8), Article el4577. 10.1111/petr.14577 37563804

