# Supplementary material for: Strengths-Based Programs for Youth at Risk for Toxic Stress: A Scoping Review of Programs Targeting Mental Health, Substance Use, Parenting Skills, and Family Functioning
Source: Trauma Violence Abuse. 2025 Mar 29;27(3):934–53. doi: 10.1177/15248380251326902 (PMC13287340; doi:10.1177/15248380251326902)
Supplement: sj-docx-1-tva-10.1177_15248380251326902 – Supplemental material for Strengths-Based Programs for Youth at Risk for Toxic Stress: A Scoping Review of Programs Targeting Mental Health, Substance Use, Parenting Skills, and Family Functioning [file sj-docx-1-tva-10.1177_15248380251326902.docx]

**Supplementary Material**

**Appendix A. Search Strategies (Databases, Dates Performed, Search Terms, and Number of Results)**

| **Database & Platforms** | **Date Performed** | **Search Strategies** |
| --- | --- | --- |
| PubMed (NLM/NCBI) | 08/11/2021 | ("Parent-Child Relations"[Mesh] OR "Parents"[Mesh] OR "Family"[Mesh:NoExp] OR "Single-Parent Family"[Mesh] OR "Family Relations"[Mesh] OR "Grandparents"[Mesh] OR parent-child OR parent-adolescent OR child-parent OR father-child OR child-father OR mother-child OR child-mother OR caregiver-child OR "family relationship*" OR "family dynamic*" OR "trusted adult*" OR dyad OR dyads OR dyadic) AND (intervention* OR program OR programs OR (resilien* AND (training OR education OR "education" [Subheading]))) AND ("Trauma and Stressor Related Disorders"[Mesh] OR "adverse childhood event*" OR "adverse childhood experience*" OR dysregulation OR hyperarousal OR hypoarousal OR adversity[tiab] OR family dysfunction* OR broken home* OR "toxic stress") AND ("Self-Control"[Mesh] OR "Self Care"[Mesh:NoExp] OR "Self Efficacy"[Mesh] OR "Resilience, Psychological"[Mesh] OR self-efficacy OR self-regulation OR co-regulation OR cognitive regulation OR emotion* regulation OR behavior* regulation OR resilience OR resilient) AND (adolescent OR adolescents OR adolescence OR teenager* OR "Adolescent Behavior"[Mesh]), Filters: English, from 2000 - 08/11/2021 |
| Medline Complete (EBSCOhost) | 08/11/2021 | ( ( MH "Parent-Child Relations+") OR (MH "Parents+") OR (MH "Family") OR (MH "Single-Parent Family") OR (MH "Family Relations+") OR (MH "Grandparents") OR ((parent OR father OR mother OR caregiver) N2 (child OR adolescent OR teen*)) OR (family N3 (relationship* OR dynamic*)) OR "trusted adult*" OR dyad OR dyads OR dyadic ) AND ( intervention* OR program OR programs OR (resilien* N3 (training OR education)) ) AND ( (MH "Trauma and Stressor Related Disorders"+) OR "adverse childhood event*" OR "adverse childhood experience*" OR dysregulation OR hyperarousal OR hypoarousal OR adversity OR (family N3 dysfunction*) OR "broken home*" OR "toxic stress" ) AND ( (MH "Self-Control") OR (MH "Self Care") OR (MH "Self Efficacy") OR (MH "Resilience, Psychological") OR self-efficacy OR self-regulation OR co-regulation OR ((cognitive OR emotion* OR behavior*) N3 regulat*) OR resilien* OR hardiness ) AND ( adolescent OR adolescents OR adolescence OR teenager* OR (MH "Adolescent Behavior+") OR (MH "Adolescent") )  Limiters - Date of Publication: 20000101-; English Language |
| CINAHL Complete (EBSCOhost) | 08/11/2021 | ( (MH "Parent-Child Relations+") OR (MH "Parents+") OR (MH "Family") OR (MH "Family Relations+") OR (MH "Grandparents") OR ((parent OR father OR mother OR caregiver) N2 (child OR adolescent OR teen*)) OR (family N3 (relationship* OR dynamic*)) OR "trusted adult*" OR dyad OR dyads OR dyadic ) AND ( intervention* OR program OR programs OR (resilien* N3 (training OR education)) OR (MH "Hardiness/ED") ) AND ( (trauma* N3 stressor-related N3 disorder*) OR "adverse childhood event*" OR "adverse childhood experience*" OR dysregulation OR hyperarousal OR hypoarousal OR adversity OR (family N3 dysfunction*) OR "broken home*" OR "toxic stress" ) AND ( (MH "Self-Control+") OR (MH "Self Care") OR (MH "Self Efficacy") OR (MH "Hardiness") OR self-efficacy OR self-regulation OR co-regulation OR ((cognitive OR emotion* OR behavior*) N3 regulat*) OR resilien* ) AND ( adolescent OR adolescents OR adolescence OR teenager* OR (MH "Adolescent Behavior") OR (MH "Adolescence") )  Limiters - Published Date: 20000101-; English Language |
| PsycINFO (ProQuest) | 08/11/2021 | (MAINSUBJECT.EXACT.EXPLODE("Parent Child Relations") OR MAINSUBJECT.EXACT.EXPLODE("Parents") OR MAINSUBJECT.EXACT("Family") OR MAINSUBJECT.EXACT.EXPLODE("Family Relations") OR MAINSUBJECT.EXACT("Grandparents") OR ((parent OR father OR mother OR caregiver) NEAR/2 (child OR adolescent OR teen*)) OR (family NEAR/3 (relationship* OR dynamic*)) OR "trusted adult*" OR MAINSUBJECT.EXACT("Dyads") OR dyad OR dyads OR dyadic) AND (intervention* OR program OR programs OR (resilien* NEAR/3 (training OR education))) AND (MAINSUBJECT.EXACT.EXPLODE("Stress and Trauma Related Disorders") OR MAINSUBJECT.EXACT("Childhood Adversity") OR "adverse childhood event*" OR "adverse childhood experience*" OR MAINSUBJECT.EXACT("Disruptive Mood Dysregulation Disorder") OR dysregulation OR hyperarousal OR hypoarousal OR adversity OR "broken home*" OR "toxic stress") AND (MAINSUBJECT.EXACT.EXPLODE("Self-Control") OR MAINSUBJECT.EXACT("Self-Care") OR MAINSUBJECT.EXACT.EXPLODE("Self-Care Skills") OR MAINSUBJECT.EXACT("Child Self Care") OR MAINSUBJECT.EXACT("Self-Efficacy") OR MAINSUBJECT.EXACT("Resilience (Psychological)") OR self-efficacy OR self-regulation OR co-regulation OR ((cognitive OR emotion* OR behavior*) NEAR/3 regulat*) OR resilien*) AND (adolescent OR adolescents OR adolescence OR teenager* OR MAINSUBJECT.EXACT("Adolescent Behavior") OR MAINSUBJECT.EXACT("Early Adolescence")) Additional limits - Date: After 1999; Language: English  Applied filters: NOT (Books AND Dissertations & Theses |
| Web of Science Core Collection (Clarivate) | 08/11/2021 | TOPIC: (grandparent* OR ((parent OR father OR mother OR caregiver) NEAR/1 (child OR adolescent OR teen*)) OR (family NEAR/3 (relationship* OR dynamic*)) OR "trusted adult*" OR dyad OR dyads OR dyadic) AND TOPIC: (intervention* OR program OR programs OR (resilien* NEAR/3 (training OR education))) AND TOPIC: (trauma* OR stress OR stressful OR stressed OF stressor OR post-traumatic OR "adverse childhood event*" OR "adverse childhood experience*" OR dysregulation OR hyperarousal OR hypoarousal OR adversity OR "broken home*") AND TOPIC: (self-control OR self-care OR self-efficacy OR self-regulation OR co-regulation OR ((cognitive OR emotion* OR behavior*) NEAR/3 regulat*) OR resilien*) AND TOPIC: (adolescent OR adolescents OR adolescence OR teenager*)  Refined by: DOCUMENT TYPES: ( ARTICLE OR REVIEW OR EARLY ACCESS ) AND LANGUAGES: ( ENGLISH )  Timespan: 2000-2021. Indexes: SCI-EXPANDED, SSCI, A&HCI. |
| Scopus (Elsevier) | 08/11/2021 | ( TITLE-ABS-KEY ( grandparent* OR ( ( parent OR father OR mother OR caregiver ) W/1 ( child OR adolescent OR teen* ) ) OR ( family W/3 ( relationship* OR dynamic* ) ) OR "trusted adult*" OR dyad OR dyads OR dyadic ) AND TITLE-ABS-KEY ( intervention* OR program OR programs OR ( resilien* W/3 ( training OR education ) ) ) AND TITLE-ABS-KEY ( trauma* OR stress OR stressful OR stressed AND of AND stressor OR post-traumatic OR "adverse childhood event*" OR "adverse childhood experience*" OR dysregulation OR hyperarousal OR hypoarousal OR adversity OR "broken home*" ) AND TITLE-ABS-KEY ( self-control OR self-care OR self-efficacy OR self-regulation OR co-regulation OR ( ( cognitive OR emotion* OR behavior* ) W/3 regulat* ) OR resilien* ) AND TITLE-ABS-KEY ( adolescent OR adolescents OR adolescence OR teenager* ) ) AND PUBYEAR > 1999 AND ( LIMIT-TO ( DOCTYPE , "ar" ) OR LIMIT-TO ( DOCTYPE , "re" ) OR LIMIT-TO ( DOCTYPE , "Undefined" ) ) AND ( LIMIT-TO ( LANGUAGE , "English" ) )  (Excluded "Conference Paper") |
| Cochrane Library | 08/12/2021 | [mh "Parent-Child Relations"] OR [mh Parents] OR [mh ^Family] OR [mh "Single-Parent Family"] OR [mh "Family Relations"] OR [mh Grandparents] OR ((parent OR father OR mother OR caregiver) NEAR/2 (child OR adolescent OR teen*)) OR (family NEAR/3 (relationship* OR dynamic*)) OR "trusted adult*" OR dyad OR dyads OR dyadic AND intervention* OR program OR programs OR (resilien* NEAR/3 (training OR education)) AND [mh "Trauma and Stressor Related Disorders"] OR "adverse childhood event*" OR "adverse childhood experience*" OR dysregulation OR hyperarousal OR hypoarousal OR adversity OR (family NEAR/3 dysfunction*) OR "broken home*" OR "toxic stress" AND [mh "Self-Control"] OR [mh ^"Self Care"] OR [mh "Self Efficacy"] OR [mh "Resilience, Psychological"] OR self-efficacy OR self-regulation OR co-regulation OR ((cognitive OR emotion* OR behavior*) NEAR/3 regulat*) OR resilien* OR hardiness AND adolescent OR adolescents OR adolescence OR teenager* OR [mh "Adolescent Behavior"] OR [mh Adolescent]  Cochrane Library publication date from Jan 2000 to 08/12/2021, in Cochrane Reviews  17 Cochrane Reviews matching "#6 - #1 AND #2 AND #3 AND #4 AND #5" Cochrane Database of Systematic Reviews Issue 8 of 12, August 2021  15 Trials matching "#6 - #1 AND #2 AND #3 AND #4 AND #5" Cochrane Central Register of Controlled Trials Issue 8 of 12, August 2021 |
| Total Number of Results Before Removing Duplicates: **1040** | | |
| **Updated Search** | | |
| PubMed Update (NLM/NCBI) | 12/13/2022 | ("Parent-Child Relations"[Mesh] OR "Parents"[Mesh] OR "Family"[Mesh:NoExp] OR "Single-Parent Family"[Mesh] OR "Family Relations"[Mesh] OR "Grandparents"[Mesh] OR parent-child OR parent-adolescent OR child-parent OR father-child OR child-father OR mother-child OR child-mother OR caregiver-child OR "family relationship*" OR "family dynamic*" OR "trusted adult*" OR dyad OR dyads OR dyadic) AND (intervention* OR program OR programs OR (resilien* AND (training OR education OR "education" [Subheading]))) AND ("Trauma and Stressor Related Disorders"[Mesh] OR "adverse childhood event*" OR "adverse childhood experience*" OR dysregulation OR hyperarousal OR hypoarousal OR adversity[tiab] OR family dysfunction* OR broken home* OR "toxic stress") AND ("Self-Control"[Mesh] OR "Self Care"[Mesh:NoExp] OR "Self Efficacy"[Mesh] OR "Resilience, Psychological"[Mesh] OR self-efficacy OR self-regulation OR co-regulation OR cognitive regulation OR emotion* regulation OR behavior* regulation OR resilience OR resilient) AND (adolescent OR adolescents OR adolescence OR teenager* OR "Adolescent Behavior"[Mesh])  Filters: English, from 2021 -12/13/2022 |
| Medline Complete Update (EBSCOhost) | 12/13/2022 | ( (MH "Parent-Child Relations+") OR (MH "Parents+") OR (MH "Family") OR (MH "Single-Parent Family") OR (MH "Family Relations+") OR (MH "Grandparents") OR ((parent OR father OR mother OR caregiver) N2 (child OR adolescent OR teen*)) OR (family N3 (relationship* OR dynamic*)) OR "trusted adult*" OR dyad OR dyads OR dyadic ) AND ( intervention* OR program OR programs OR (resilien* N3 (training OR education)) ) AND ( (MH "Trauma and Stressor Related Disorders+") OR "adverse childhood event*" OR "adverse childhood experience*" OR dysregulation OR hyperarousal OR hypoarousal OR adversity OR (family N3 dysfunction*) OR "broken home*" OR "toxic stress" ) AND ( (MH "Self-Control+") OR (MH "Self Care") OR (MH "Self Efficacy") OR (MH "Resilience, Psychological") OR self-efficacy OR self-regulation OR co-regulation OR ((cognitive OR emotion* OR behavior*) N3 regulat*) OR resilien* OR hardiness ) AND ( adolescent OR adolescents OR adolescence OR teenager* OR (MH "Adolescent Behavior+") OR (MH "Adolescent") ) Limiters - Date of Publication: 20210101-; English Language |
| CINAHL Complete Update (EBSCOhost) | 12/13/2022 | ( (MH "Parent-Child Relations+") OR (MH "Parents+") OR (MH "Family") OR (MH "Family Relations+") OR (MH "Grandparents") OR ((parent OR father OR mother OR caregiver) N2 (child OR adolescent OR teen*)) OR (family N3 (relationship* OR dynamic*)) OR "trusted adult*" OR dyad OR dyads OR dyadic ) AND ( intervention* OR program OR programs OR (resilien* N3 (training OR education)) OR (MH "Hardiness/ED") ) AND ( (trauma* N3 stressor-related N3 disorder*) OR "adverse childhood event*" OR "adverse childhood experience*" OR dysregulation OR hyperarousal OR hypoarousal OR adversity OR (family N3 dysfunction*) OR "broken home*" OR "toxic stress" ) AND ( (MH "Self-Control+") OR (MH "Self Care") OR (MH "Self Efficacy") OR (MH "Hardiness") OR self-efficacy OR self-regulation OR co-regulation OR ((cognitive OR emotion* OR behavior*) N3 regulat*) OR resilien ) AND ( adolescent OR adolescents OR adolescence OR teenager* OR (MH "Adolescent Behavior") OR (MH "Adolescence") )  Limiters - Date of Publication: 20210101-; English Language |
| PsycINFO Update (ProQuest) | 12/13/2022 | (MAINSUBJECT.EXACT.EXPLODE("Parent Child Relations") OR MAINSUBJECT.EXACT.EXPLODE("Parents") OR MAINSUBJECT.EXACT("Family") OR MAINSUBJECT.EXACT.EXPLODE("Family Relations") OR MAINSUBJECT.EXACT("Grandparents") OR ((parent OR father OR mother OR caregiver) NEAR/2 (child OR adolescent OR teen*)) OR (family NEAR/3 (relationship* OR dynamic*)) OR "trusted adult*" OR MAINSUBJECT.EXACT("Dyads") OR dyad OR dyads OR dyadic) AND (intervention* OR program OR programs OR (resilien* NEAR/3 (training OR education))) AND (MAINSUBJECT.EXACT.EXPLODE("Stress and Trauma Related Disorders") OR MAINSUBJECT.EXACT("Childhood Adversity") OR "adverse childhood event*" OR "adverse childhood experience*" OR MAINSUBJECT.EXACT("Disruptive Mood Dysregulation Disorder") OR dysregulation OR hyperarousal OR hypoarousal OR adversity OR "broken home*" OR "toxic stress") AND (MAINSUBJECT.EXACT.EXPLODE("Self-Control") OR MAINSUBJECT.EXACT("Self-Care") OR MAINSUBJECT.EXACT.EXPLODE("Self-Care Skills") OR MAINSUBJECT.EXACT("Child Self Care") OR MAINSUBJECT.EXACT("Self-Efficacy") OR MAINSUBJECT.EXACT("Resilience (Psychological)") OR self-efficacy OR self-regulation OR co-regulation OR ((cognitive OR emotion* OR behavior*) NEAR/3 regulat*) OR resilien*) AND (adolescent OR adolescents OR adolescence OR teenager* OR MAINSUBJECT.EXACT("Adolescent Behavior") OR MAINSUBJECT.EXACT("Early Adolescence")) Additional limits - Date: After 2020; Language: English Applied filters: NOT (Books AND Dissertations & Theses) |
| Web of Science Core Collection Update (Clarivate) | 12/13/2022 | grandparent* OR ((parent OR father OR mother OR caregiver) NEAR/1 (child OR adolescent OR teen*)) OR (family NEAR/3 (relationship* OR dynamic*)) OR "trusted adult*" OR dyad OR dyads OR dyadic (Topic) and intervention* OR program OR programs OR (resilien* NEAR/3 (training OR education)) (Topic) and trauma* OR stress OR stressful OR stressed OF stressor OR post-traumatic OR "adverse childhood event*" OR "adverse childhood experience*" OR dysregulation OR hyperarousal OR hypoarousal OR adversity OR "broken home*" (Topic) and self-control OR self-care OR self-efficacy OR self-regulation OR co-regulation OR ((cognitive OR emotion* OR behavior*) NEAR/3 regulat*) OR resilien* (Topic) and adolescent OR adolescents OR adolescence OR teenager* (Topic) and Article or Early Access or Review Article (Document Types) and Book Chapters or Proceeding Paper (Exclude – Document Types) and English (Languages) and Emerging Sources Citation Index (ESCI) (Exclude – Web of Science Index), Timespan: 2021-01-01 to 2023-12-31. Indexes: SCI-EXPANDED, SSCI. |
| Scopus Update (Elsevier) | 12/13/2022 | ( TITLE-ABS-KEY ( grandparent* OR ( ( parent OR father OR mother OR caregiver ) W/1 ( child OR adolescent OR teen* ) ) OR ( family W/3 ( relationship* OR dynamic* ) ) OR "trusted adult*" OR dyad OR dyads OR dyadic ) AND TITLE-ABS-KEY ( intervention* OR program OR programs OR ( resilien* W/3 ( training OR education ) ) ) AND TITLE-ABS-KEY ( trauma* OR stress OR stressful OR stressed AND of AND stressor OR post-traumatic OR "adverse childhood event*" OR "adverse childhood experience*" OR dysregulation OR hyperarousal OR hypoarousal OR adversity OR "broken home*" ) AND TITLE-ABS-KEY ( self-control OR self-care OR self-efficacy OR self-regulation OR co-regulation OR ( ( cognitive OR emotion* OR behavior* ) W/3 regulat* ) OR resilien* ) AND TITLE-ABS-KEY ( adolescent OR adolescents OR adolescence OR teenager* ) ) AND PUBYEAR > 2020 AND ( LIMIT-TO ( DOCTYPE , "ar" ) OR LIMIT-TO ( DOCTYPE , "re" ) ) AND ( LIMIT-TO ( LANGUAGE , "English" ) ) |
| Cochrane Library Update | 12/13/2022 | [mh "Parent-Child Relations"] OR [mh Parents] OR [mh ^Family] OR [mh "Single-Parent Family"] OR [mh "Family Relations"] OR [mh Grandparents] OR ((parent OR father OR mother OR caregiver) NEAR/2 (child OR adolescent OR teen*)) OR (family NEAR/3 (relationship* OR dynamic*)) OR "trusted adult*" OR dyad OR dyads OR dyadic AND  intervention* OR program OR programs OR (resilien* NEAR/3 (training OR education)) AND  [mh "Trauma and Stressor Related Disorders"] OR "adverse childhood event*" OR "adverse childhood experience*" OR dysregulation OR hyperarousal OR hypoarousal OR adversity OR (family NEAR/3 dysfunction*) OR "broken home*" OR "toxic stress" AND  [mh "Self-Control"] OR [mh ^"Self Care"] OR [mh "Self Efficacy"] OR [mh "Resilience, Psychological"] OR self-efficacy OR self-regulation OR co-regulation OR ((cognitive OR emotion* OR behavior*) NEAR/3 regulat*) OR resilien* OR hardiness AND  adolescent OR adolescents OR adolescence OR teenager* OR [mh "Adolescent Behavior"] OR [mh Adolescent]    1 Cochrane Review matching "#6 - #1 AND #2 AND #3 AND #4 AND #5" with Cochrane Library publication date Between Jan 2021 and Dec 2022, in Cochrane Reviews  Cochrane Database of Systematic Reviews Issue 12 of 12, December 2022  17 Trials matching "#6 - #1 AND #2 AND #3 AND #4 AND #5" with Publication Year from 2021 to 2022, with Cochrane Library publication date Between Jan 2021 and Dec 2022, in Trials (Word variations have been searched)  Cochrane Central Register of Controlled Trials Issue 11 of 12, November 2022 |
| Total Number of Results Before Removing Duplicates: **332** | | |
| **Updated Search (manual search based on programs’ name in excluded articles)** | | |
| PubMed, Medline, CINAHL, Web of Science, Scopus, PsycINFO, Cochrane Library, and Google Scholar | 02/25/2023 | "Multidimensional Treatment Foster Care" AND ("family intervention" OR "family therapy" OR "family counseling" OR "family support") AND (trauma OR PTSD OR "post-traumatic stress" OR "psychological trauma") AND (adolescents OR teens OR youths)  "ATraPA" AND ("family intervention" OR "family therapy" OR "family counseling" OR "family support") AND (trauma OR PTSD OR "post-traumatic stress" OR "psychological trauma") AND (adolescents OR teens OR youths)  "Overcoming Under Stress" AND ("family intervention" OR "family therapy" OR "family counseling" OR "family support") AND (trauma OR PTSD OR "post-traumatic stress" OR "psychological trauma") AND (adolescents OR teens OR youths)  "UCLA Trauma Grief " AND ("family intervention" OR "family therapy" OR "family counseling" OR "family support") AND (trauma OR PTSD OR "post-traumatic stress" OR "psychological trauma") AND (adolescents OR teens OR youths)  "Project Talk" AND ("family intervention" OR "family therapy" OR "family counseling" OR "family support") AND (trauma OR PTSD OR "post-traumatic stress" OR "psychological trauma") AND (adolescents OR teens OR youths)  "Project Family Talk" AND ("family intervention" OR "family therapy" OR "family counseling" OR "family support") AND (trauma OR PTSD OR "post-traumatic stress" OR "psychological trauma") AND (adolescents OR teens OR youths)  "Triple P" AND ("family intervention" OR "family therapy" OR "family counseling" OR "family support") AND (trauma OR PTSD OR "post-traumatic stress" OR "psychological trauma") AND (adolescents OR teens OR youths)  "Child and Family Traumatic Stress" AND ("family intervention" OR "family therapy" OR "family counseling" OR "family support") AND (trauma OR PTSD OR "post-traumatic stress" OR "psychological trauma") AND (adolescents OR teens OR youths)  "Care Process Model" AND ("family intervention" OR "family therapy" OR "family counseling" OR "family support") AND (trauma OR PTSD OR "post-traumatic stress" OR "psychological trauma") AND (adolescents OR teens OR youths)  "Trauma-Focused Cognitive Behavioral Therapy" AND ("family intervention" OR “family counseling" OR "family support") AND (trauma OR PTSD OR "post-traumatic stress" OR "psychological trauma") AND (adolescents OR teens OR youths)  "Cognitive Behavioral Therapy" AND ("family intervention" OR "family therapy" OR "family counseling" OR "family support") AND (trauma OR PTSD OR "post-traumatic stress" OR "psychological trauma") AND (adolescents OR teens OR youths)  "Combined Parent-Child Cognitive-Behavioral Therapy" AND ("family intervention" OR "family therapy" OR "family counseling" OR "family support") AND (trauma OR PTSD OR "post-traumatic stress" OR "psychological trauma") AND (adolescents OR teens OR youths)  Filters: English, from 2000 - 02/25/2023 |
| Total Number of Results: 0 | | |
| **Updated Search** | | |
| PubMed (NLM/NCBI) | 1/19/2024 | ("Parent-Child Relations"[Mesh] OR "Parents"[Mesh] OR "Family"[Mesh:NoExp] OR "Single-Parent Family"[Mesh] OR "Family Relations"[Mesh] OR "Grandparents"[Mesh] OR parent-child OR parent-adolescent OR child-parent OR father-child OR child-father OR mother-child OR child-mother OR caregiver-child OR "family relationship*" OR "family dynamic*" OR "trusted adult*" OR dyad OR dyads OR dyadic) AND (intervention* OR program OR programs OR (resilien* AND (training OR education OR "education" [Subheading]))) AND ("Trauma and Stressor Related Disorders"[Mesh] OR "adverse childhood event*" OR "adverse childhood experience*" OR dysregulation OR hyperarousal OR hypoarousal OR adversity[tiab] OR family dysfunction* OR broken home* OR "toxic stress") AND ("Self-Control"[Mesh] OR "self Care"[Mesh:NoExp] OR "Self Efficacy"[Mesh] OR "Resilience, Psychological"[Mesh] OR self-efficacy OR self-regulation OR co-regulation OR cognitive regulation OR emotion* regulation OR behavior* regulation OR resilience OR resilient) AND (adolescent OR adolescents OR adolescence OR teenager* OR "Adolescent Behavior"[Mesh])  Filters: English, from 2022 - 3000/12/12 |
| Medline Complete (EBSCOhost) | 1/19/2024 | ( (MH "Parent-Child Relations+") OR (MH "Parents+") OR (MH "Family") OR (MH "Single-Parent Family") OR (MH "Family Relations+") OR (MH "Grandparents") OR ((parent OR father OR mother OR caregiver) N2 (child OR adolescent OR teen*)) OR (family N3 (relationship* OR dynamic*)) OR "trusted adult*" OR dyad OR dyads OR dyadic ) AND ( intervention* OR program OR programs OR (resilien* N3 (training OR education)) ) AND ( (MH "Trauma and Stressor Related Disorders+") OR "adverse childhood event*" OR "adverse childhood experience*" OR dysregulation OR hyperarousal OR hypoarousal OR adversity OR (family N3 dysfunction*) OR "broken home*" OR "toxic stress" ) AND ( (MH "Self-Control+") OR (MH "Self Care") OR (MH "Self Efficacy") OR (MH "Resilience, Psychological") OR self-efficacy OR self-regulation OR co-regulation OR ((cognitive OR emotion* OR behavior*) N3 regulat*) OR resilien* OR hardiness ) AND ( adolescent OR adolescents OR adolescence OR teenager* OR (MH "Adolescent Behavior+") OR (MH "Adolescent") )  Limiters - Publication Date: 20220101-; English Language |
| CINAHL Complete (EBSCOhost) | 1/19/2024 | ( (MH "Parent-Child Relations+") OR (MH "Parents+") OR (MH "Family") OR (MH "Family Relations+") OR (MH "Grandparents") OR ((parent OR father OR mother OR caregiver) N2 (child OR adolescent OR teen*)) OR (family N3 (relationship* OR dynamic*)) OR "trusted adult*" OR dyad OR dyads OR dyadic ) AND ( intervention* OR program OR programs OR (resilien* N3 (training OR education)) OR (MH "Hardiness/ED") ) AND ( (trauma* N3 stressor-related N3 disorder*) OR "adverse childhood event*" OR "adverse childhood experience*" OR dysregulation OR hyperarousal OR hypoarousal OR adversity OR (family N3 dysfunction*) OR "broken home*" OR "toxic stress" ) AND ( (MH "Self-Control+") OR (MH "Self Care") OR (MH "Self Efficacy") OR (MH "Hardiness") OR self-efficacy OR self-regulation OR co-regulation OR ((cognitive OR emotion* OR behavior*) N3 regulat*) OR resilien* ) AND ( adolescent OR adolescents OR adolescence OR teenager* OR (MH "Adolescent Behavior") OR (MH "Adolescence") )  Limiters - Date of Publication: 20220101-; English Language |
| PsycINFO (ProQuest) | 1/19/2024 | (MAINSUBJECT.EXACT.EXPLODE("Parent Child Relations") OR MAINSUBJECT.EXACT.EXPLODE("Parents") OR MAINSUBJECT.EXACT("Family") OR MAINSUBJECT.EXACT.EXPLODE("Family Relations") OR MAINSUBJECT.EXACT("Grandparents") OR ((parent OR father OR mother OR caregiver) NEAR/2 (child OR adolescent OR teen*)) OR (family NEAR/3 (relationship* OR dynamic*)) OR "trusted adult*" OR MAINSUBJECT.EXACT("Dyads") OR dyad OR dyads OR dyadic) AND (intervention* OR program OR programs OR (resilien* NEAR/3 (training OR education))) AND (MAINSUBJECT.EXACT.EXPLODE("Stress and Trauma Related Disorders") OR MAINSUBJECT.EXACT("Childhood Adversity") OR "adverse childhood event*" OR "adverse childhood experience*" OR MAINSUBJECT.EXACT("Disruptive Mood Dysregulation Disorder") OR dysregulation OR hyperarousal OR hypoarousal OR adversity OR "broken home*" OR "toxic stress") AND (MAINSUBJECT.EXACT.EXPLODE("Self-Control") OR MAINSUBJECT.EXACT("Self-Care") OR MAINSUBJECT.EXACT.EXPLODE("Self-Care Skills") OR MAINSUBJECT.EXACT("Child Self Care") OR MAINSUBJECT.EXACT("Self-Efficacy") OR MAINSUBJECT.EXACT("Resilience (Psychological)") OR self-efficacy OR self-regulation OR co-regulation OR ((cognitive OR emotion* OR behavior*) NEAR/3 regulat*) OR resilien*) AND (adolescent OR adolescents OR adolescence OR teenager* OR MAINSUBJECT.EXACT("Adolescent Behavior") OR MAINSUBJECT.EXACT("Early Adolescence"))  Additional limits - Date: After 2021; Language: English  Applied filters: NOT (Books AND Dissertations & Theses) |
| Web of Science Core Collection (Clarivate) | 1/19/2024 | grandparent* OR ((parent OR father OR mother OR caregiver) NEAR/1 (child OR adolescent OR teen*)) OR (family NEAR/3 (relationship* OR dynamic*)) OR "trusted adult*" OR dyad OR dyads OR dyadic (Topic) and intervention* OR program OR programs OR (resilien* NEAR/3 (training OR education)) (Topic) and trauma* OR stress OR stressful OR stressed OR stressor OR post-traumatic OR "adverse childhood event*" OR "adverse childhood experience*" OR dysregulation OR hyperarousal OR hypoarousal OR adversity OR "broken home*" (Topic) and self-control OR self-care OR self-efficacy OR self-regulation OR co-regulation OR ((cognitive OR emotion* OR behavior*) NEAR/3 regulat*) OR resilien* (Topic) and adolescent OR adolescents OR adolescence OR teenager* (Topic) and Article or Early Access or Review Article (Document Types) and English (Languages) and Emerging Sources Citation Index (ESCI) (Exclude – Web of Science Index) and Social Sciences Citation Index (SSCI) or Science Citation Index Expanded (SCI-EXPANDED) (Web of Science Index)  Timespan: 2022-01-01 to 2024-12-31. Indexes: SCI-EXPANDED, SSCI.  Databases included: Science Citation Index Expanded (SCI-EXPANDED) --1900-present, Social Sciences Citation Index (SSCI) --1900-present. Databases Excluded: Emerging Sources Citation Index |
| Scopus (Elsevier) | 1/19/2024 | ( TITLE-ABS-KEY ( grandparent* OR ( ( parent OR father OR mother OR caregiver ) W/1 ( child OR adolescent OR teen* ) ) OR ( family W/3 ( relationship* OR dynamic* ) ) OR "trusted adult*" OR dyad OR dyads OR dyadic ) AND TITLE-ABS-KEY ( intervention* OR program OR programs OR ( resilien* W/3 ( training OR education ) ) ) AND TITLE-ABS-KEY ( trauma* OR stress OR stressful OR stressed OR stressor OR post-traumatic OR "adverse childhood event*" OR "adverse childhood experience*" OR dysregulation OR hyperarousal OR hypoarousal OR adversity OR "broken home*" ) AND TITLE-ABS-KEY ( self-control OR self-care OR self-efficacy OR self-regulation OR co-regulation OR ( ( cognitive OR emotion* OR behavior* ) W/3 regulat* ) OR resilien* ) AND TITLE-ABS-KEY ( adolescent OR adolescents OR adolescence OR teenager* ) ) |
| Cochrane Library | 1/19/2024 | [mh "Parent-Child Relations"] OR [mh Parents] OR [mh ^Family] OR [mh "Single-Parent Family"] OR [mh "Family Relations"] OR [mh Grandparents] OR ((parent OR father OR mother OR caregiver) NEAR/2 (child OR adolescent OR teen*)) OR (family NEAR/3 (relationship* OR dynamic*)) OR (trusted NEAR/2 adult*) OR dyad OR dyads OR dyadic  AND  intervention* OR program OR programs OR (resilien* NEAR/3 (training OR education))  AND  [mh "Trauma and Stressor Related Disorders"] OR "adverse childhood event" OR "adverse childhood events" OR "adverse childhood experience" OR "adverse childhood experiences" OR dysregulation OR hyperarousal OR hypoarousal OR adversity OR (family NEAR/3 dysfunction*) OR (broken NEAR/2 home*) OR (toxic NEAR/3 stress*)  AND  [mh "Self-Control"] OR [mh ^"Self Care"] OR [mh "Self Efficacy"] OR [mh "Resilience, Psychological"] OR self-efficacy OR self-regulation OR co-regulation OR ((cognitive OR emotion* OR behavior*) NEAR/3 regulat*) OR resilien* OR hardiness  AND  adolescent OR adolescents OR adolescence OR teenager* OR [mh "Adolescent Behavior"] OR [mh Adolescent]  #1 AND #2 AND #3 AND #4 AND #5"  7 Cochrane Review matching "#6 - #1 AND #2 AND #3 AND #4 AND #5" with Cochrane Library publication date from Jan 2022 to Dec 2024, in Cochrane Reviews  Cochrane Database of Systematic Reviews  Issue 1 of 12, January 2024  13 Trials matching "#6 - #1 AND #2 AND #3 AND #4 AND #5" with Publication Year from 2022 to 2024, with Cochrane Library publication date Between Jan 2022 and Dec 2024, in Trials  Cochrane Central Register of Controlled Trials  Issue 1 of 12, January 2024 |
| Total Number of Results Before Removing Duplicates: 1249 | | |
| **Updated Search (manual search based on programs’ name in excluded articles)** | | |
| PubMed, Medline, CINAHL, Web of Science, Scopus, PsycINFO, Cochrane Library, and Google Scholar | 1/30/2024 | Evidence-based behavioral parent training (BPT)  Mindfulness Training  Teaching, Raising, and Communicating with Kids (TRACK) intervention program  Family Management Efficacy (FAME)  The Family Bereavement Program  Parent Connectors program  Child and Family Traumatic Stress Intervention (CFTSI)  FOCUS (Families Over Coming Under Stress)  Care Process Model for Pediatric Traumatic Stress (CPM-PTS)  Trauma-Focused Cognitive Behavioral Therapy (TF-CBT)  Alternatives for Families: A Cognitive Behavioral Therapy (AF-CBT)  Combined Parent Child Cognitive-Behavioral Therapy (CPC-CBT)  Family Resilience-Oriented Training and Services (Recover from Crisis, Trauma, and Loss)  Family adaptation to complicated, traumatic loss  Navigate Disruptive Family Transitions Divorce & stepfamily adaptation  Overcome Obstacles to Success: At-risk Youth Child and Adolescent Developmental Challenges  Family—School Partnership Program  Gang Reduction/Youth Development (GRYD)  Preparing for the Drug-Free Years  Parents Who Care  Staying connected with Your Teen  GREAT Families  Strengthening Families Program  Family Check-Up  RELAX - Regulating Emotions Like An eXpert  VUKA Family Program  Home-based Sensory Interventions  Physical Exercise and Virtual Training  Rational-emotive language education (RELE program)  Multimodal Anxiety and Social Skills Intervention (MASSI)  Positive behavior support (PBS) practices |
| Total Number of Results Before Removing Duplicates: 2 | | |

**Appendix B. Study Characteristics**

| **No.** | **Authors & Publication Date** | **Design (RCT: randomized control trial,**  **NE:non/quasi experimental, MM: mixed, Q: qual)** | | | | **Sample Size** | | | **Measurements/ Tools** | | **Key Findings** | **Limitations** |
| --- | --- | --- | --- | --- | --- | --- | --- | --- | --- | --- | --- | --- |
|  |  | **RCT** | **EN** | **MM** | **Q** | **Families** | **Youth** | **Caregivers** | **Target** |  |  |  |
| **2** | **[Wijesekera](https://onlinelibrary.wiley.com/doi/10.1111/petr.14577)**  **2023** | **✓** |  |  |  | **17**  **Families** | **17** | **17** | **MH**  **FF/PS** | **-Pediatric Quality of Life Inventory**  **(PedsQL)**  **-Strengths and Difficulties**  **Questionnaire (SDQ)**  **-Moods and Feelings Questionnaire,**  **Short Form (MFQ)**  **-Screen for Child Anxiety Related**  **Emotional Disorders (SCARED)**  **-Child Trauma Screening**  **Questionnaire (CTSQ)**  **-Patient Health Questionnaire (PHQ-8) -Generalized Anxiety Disorder**  **(GAD-7)**  **-Posttraumatic Stress Disorder**  **Checklist, Civilian Version (PCL-C)  -McMaster Family Assessment Device**  **(FAD)**  **-Adolescent Medication Barriers Scale**  **(AMBS)**  **-Parent Medication Barriers Scale**  **(PMBS)** | **- Significant reductions in adolescents' internalizing behavior (p = 0.0235).**  **- Significant reductions in parents' depression (p = 0.0313) and post-traumatic stress symptoms (p= 0.0313). - No significant changes in parent reports of medication adherence barriers or family functioning.** | **- Unexpected illnesses. - Hospitalizations related to the ill child. - Busy parent work schedules. - Small sample size.** |
| **4** | [**Taliercio**](https://pubmed.ncbi.nlm.nih.gov/37363718/)  **2023** |  | **✓** |  |  | **35** | **35** |  | **MH**  **FF/PS** | **-Patient Health Questionnaire 9 (PHQ-9) -Generalized Anxiety Disorder-7 (GAD-7) -Sheehan Disability Scale (SDS) -Difficulties in Emotion Regulation Scale 16 (DERS-16) -DSeliberate Self-Harm Inventory (DSHI)  - No specific measurements/tools reported** | **- Significant reduction in depression (p<0.01, ηp2=0.51), anxiety (p<0.01, ηp2=0.40), and self-harm behavior (p=0.02, φ=0.59) from intake to the 3-month follow-up; significant improvement in daily functioning (p=0.02, ηp2=0.84) and emotion regulation (p=0.01, ηp2=0.39) in youth. - Skills and support offered in a parent-only group to both normalize parental difficulties and reduce shame.** | **- No control group or randomization. - Sample size changed throughout assessments due to attrition. - No assessment of treatment fidelity. - Self-reported data. - Confounding variables such as parental engagement and the impact of COVID-19.** |
| **5** | [**Loo**](https://pubmed.ncbi.nlm.nih.gov/36930214/)  [**2023**](https://pubmed.ncbi.nlm.nih.gov/36930214/) |  | **✓** |  |  | **703** | **703** |  | **MH**  **FF/PS** | **-Pediatric Symptom Checklist-17 (PSC-17) -Caregiver Strain Questionnaire-Short Form 7 (CGSQ-SF7) -Generalized Anxiety Disorder 7-item (GAD-7) -Patient Health Questionnaire 8-item (PHQ-8)  -Caregiver Strain Questionnaire-Short Form 7 (CGSQ-SF7)** | **- Significant improvements in therapy and tele-behavioral health coaching participants' overall psychosocial functioning, internalizing behaviors, anxiety symptoms, and depressive symptoms among children(p<0.001). - Improvement in caregiver strain (p<0.001) and significantly reduced stress associated with caring for children with behavioral health needs by caregivers.** | **- No control group. - Sample population is not representative (mostly white). - No long-term follow up data.** |
| **6** | [**Clauss**](https://pubmed.ncbi.nlm.nih.gov/36369940/)  **2023** |  |  | **✓** |  | **28** | **28** |  | **MH**    **FF/PS** | **-Strengths and Difficulties Questionnaire (SDQ) -Pediatric Symptom Checklist (PSC) -Child Behavior Checklist (CBCL)  - No specific measurements/tools reported** | **- Significant reduction in anxious/depressed symptoms from baseline (M=55.8) to post-intervention (M=52.7) (p=0.03); significant reduction in social problems from baseline (M=57.2) to post-intervention (M=53.6) (p=0.049); increased positive attribution bias in adolescents from baseline (3.6%) to post-intervention (7.8%), being more likely to label neutral faces as happy (p=0.034). - Learning new ways to understand their children's emotions, communicate with them, and regulate their emotions during challenging parenting situations by parents.** | **- All participants from the three cohorts not screened using the same instrument. - 43% of eligible families unreachable via phone or email post-screening. - Enrollment in cohort 3 affected by the COVID 19 pandemic.** |
| **7** | [**Kaufman**](https://pubmed.ncbi.nlm.nih.gov/35611597/)  [**2023**](https://pubmed.ncbi.nlm.nih.gov/35611597/) |  | **✓** |  |  | **60** | **60** | **60** | **MH**  **FF/PS** | **-The Lifetime Suicide Attempt Self-injury Interview (L-SASI) -The respiratory sinus arrhythmia (RSA) test was collected as a physiological index of emotion regulation. -An observational coding system to measure skill-related behavior across four key domains of the GIVE (be Gentle, act Interested, Validate, and use an Easy manner)  -The Issues Checklist for identifying prompts for the discussion tasks between mothers and daughters** | **- Significant improvement in GIVE skill use across the sample (B = 0.350, p = 0.004); significant improvement in "be gentle" than controls in self-injuring (B = 0.626, p < 0.001); better physiological regulation. - Improved family functioning.** | **- Small sample size. - Not able to determine whether participants had learned GIVE before participation.** |
| **8** | [**Stover**](https://pubmed.ncbi.nlm.nih.gov/36152531/)  **2022** |  | **✓** |  |  | **1190** | **1190** | **1190** | **MH**    **FF/PS** | **-Child PTSD Symptom Scale (CPSS) -UCLA PTSD Reaction Index (PTSD-RI) for DSM-IV -Posttraumatic Checklist-Civilian Version (PCL-C)  -Posttraumatic Checklist-Civilian Version (PCL-C)** | **- Significant reduction in PTSD/trauma symptoms (B = -10.24, p < .000) from pre- to post-CFTSI; 65% less likely to have a full PTSD diagnosis and 73% less likely to have partial or full PTSD compared to the control group after 3 months post-treatment. - No specific family functioning/ parenting skills were reported.** | **-Non-experimental design without control group. -Relied solely on self-report measures from children and caregivers. -No clinician ratings or diagnoses. -Did not assess acceptability, feasibility, or differences between treatment completers and non-completers. -Sample primarily composed of children who experienced sexual abuse. -Used measures based on DSM-IV criteria for PTSD, not updated DSM-5. -No direct observation or coding of sessions for fidelity monitoring.** |
| **9** | [**Cloutier**](https://pubmed.ncbi.nlm.nih.gov/34498386/)  **2022** |  | **✓** |  |  |  | **46** |  | **MH**  **FF/PS** | **-Suicide Ideation Questionnaire, Junior (SIQ-JR) -Children's Depression Inventory-2 Self-Report (CDI-2 SR) -Multidimensional Anxiety Scale for Children 2nd Edition (MASC-2) -The Perceived Stress Scale (PSS)  -Inventory of Parent and Peer Attachment (IPPA) -Relationship Scale Questionnaire (RSQ)** | **- Significant reductions in adolescents' depression (pre-post difference = 9.3, d = 0.70, p = .001), anxiety (pre-post difference = 4.5, d = 0.54, p = .006), suicidal ideation (pre-post difference = 18.1, d = 0.82, p < .001), and perceived stress (pre-post difference = 7.1, d = 1.10, p < .001). - Significant decrease in caregiver-perceived stress (pre-post difference = 2.7, d = 0.42, p = .029) and caregiver-avoidant attachment style (pre-post difference = 1.6, d = 0.45, p = .019). - No significant changes in attachment security between adolescents and their caregivers.** | **- High attrition bias due to 1/3 of the sample not completing the study. - No follow-up assessment. - Small sample size.** |
| **10** | [**Neville**](https://www.ncbi.nlm.nih.gov/pmc/articles/PMC9566609/)  **2022** |  |  | **✓** |  | **257** |  |  | **MH**  **FF/PS** | **-Center for Epidemiologic Studies Depression Scale for Children (CESD-C) -WHO Disability Assessment Schedule for Children 2.0 (WHODAS-Child) -UCLA PTSD Reaction Index -Achenbach Youth Self Report (YSR) -Child Behavior Checklist (CBCL)  -Alabama Parenting Questionnaire (APQ) -Intergenerational Congruence in Immigrant Families Scale** | **- Bhutanese families' greater emphasis on child mental health (MH) improvements: significant reductions in depression (β = −9.20, p = 0.04) and conduct problems (β = −0.92, p = 0.01), less anxiety and shyness in Bhutanese children; no significant difference in trauma symptoms for both groups; less discussion of MH impacts from Somali Bantu families. - Improved family communication, positive parenting, and family time together after the FSI-R.** | **- Small quantitative Bhutanese sample. - Interview protocol limitations.** |
| **11** | [**Lu**](https://journals.sagepub.com/doi/10.1177/10497315221089684)  [**2022**](https://journals.sagepub.com/doi/10.1177/10497315221089684) |  |  | **✓** |  | **21** |  |  | **MH**  **FF/PS** | **-Parenting Stress Index Short Form -Child and Adolescent Mindfulness Measure -Strengths and Difficulties Questionnaire (SDQ)  -Chinese version of Interpersonal Mindfulness in Parenting scale -Inventory of Parents and Peer Attachment-Revised (parent attachment subscale)** | **- Significant decrease in parenting stress (partial η2 = .423, p = .02) within-group, but not in controls (partial η2 = .000). - No significant changes in mindful parenting, child mindfulness levels, child behavioral problems, or parent-child relationship in either the intervention or control group.** | **- Small sample size. - Non-randomized design. - Limited geographic scope. - Unstructured child intervention. - Short follow-up period. - Reliance on self-report measures. - Potential expectancy effects. - Limited home practice adherence. - Lack of behavioral observation. - Uncontrolled confounding factors.** |
| **13** | [**Lozano**](https://pubmed.ncbi.nlm.nih.gov/34735168/)  **2022** |  |  |  | **✓** | **12** | **12** | **12** |  | **- No specific measurements/tools reported** | **- Significant reductions in depressive symptoms, substance use, and increased condom use among LGBTQ youth.  - Increased parent support/acceptance of LGBTQ youth and significant improvement in family functioning and communication.** | **- Small sample size. - Limited geographic diversity. - Self-selection bias.** |
| **14** | [**Siebelink**](https://pubmed.ncbi.nlm.nih.gov/34030214/)  **2022** | **✓** |  |  |  | **103** |  |  | **MH**  **FF/PS** | **-Behavior Rating Inventory of Executive Function (BRIEF) -Conners' Parent/Teacher Rating Scale (CPRS/CTRS) -Strengths and Weaknesses of ADHD symptoms and Normal Behavior Scale (SWAN) -Social Responsiveness Scale -ADHD DSM-IV Rating Scale (ARS) -Depression Anxiety Stress Scale -Ruminative Response Scale (Brooding subscale) -World Health Organization Well-Being Index (WHO-5) -Mental Health Continuum-Short Form (MHC-SF)  -Self-Compassion Scale (short version) -Interpersonal Mindfulness in Parenting scale** | **- Significant improvements in ADHD symptoms, especially hyperactivity-impulsivity (post-treatment: d=0.72, p<.001; 6-month follow-up: d=0.37, p<.10), depression, anxiety, and stress (6-month follow-up: d=0.42, p<.05) compared to controls. - Significant improvements in quality of life (post-treatment: d=0.55, p<.01), mindful parenting (post-treatment: d=0.58, p<.01; 6-month follow-up: d=0.45, p<.05), and self-compassion (6-month follow-up: d=0.66, p<.01); better self-control in children at post-treatment (not statistically significant).** | **- Participants not blinded to treatment conditions. - Most outcomes are based on subjective rating scales.** |
| **16** | [**Breaux**](https://www.tandfonline.com/doi/full/10.1080/23794925.2021.1970053)  **2021** |  |  | **✓** |  | **32** | **32** | **32** | **MH**  **FF/PS** | **-Difficulties in Emotion Regulation Scale (DERS)  -Coping with Children's Negative Emotions Scale -Conflict Behavior Questionnaire** | **- Large improvements for caregivers and clinician ratings of adolescent emotion dysregulation (η2 = .18−.48) and family conflict (η2 = .26 and .43). - Moderate decreases in non-supportive emotion socialization (η2 = .11); small improvements for caregiver emotion dysregulation**  **(η2 =.03).** | **- Pilot studies, no control groups. - Delivered by intervention developers and supervised graduate clinicians. - No observational/psychophysiological ED and emotion socialization data for telehealth. - Demographic differences between in-person and telehealth samples. - Telehealth during COVID-19 pandemic.** |
| **17** | [**Li**](https://pubmed.ncbi.nlm.nih.gov/33575977/)  [**2021**](https://pubmed.ncbi.nlm.nih.gov/33575977/) |  | **✓** |  |  |  | **32** | **27** | **MH**  **FF/PS** | **-Mindful Attention Awareness Scale (MAAS) -Mindful Attention Awareness Scale for Adolescents (MAAS-A) -Perceived Stress Scale (PSS-10): -Brief Difficulties in Emotion Regulation Scale (DERS)  -Multidimensional Scale of Perceived Social Support - Family Subscale (MSPSS-Family)** | **- Significant improvement in parents' perceived stress (B= −2.94, p=0.029) and adolescents' emotional regulation (p=0.017). - Reported greater improvements in perceived social support from family (MSPSS-Family) compared to the control group (B=2.32, p=0.027) by children.** | **- Limited minority representation in population. - Potential linguistic or cultural incongruence. - Small sample size.** |
| **18** | [**Lavner**](https://pubmed.ncbi.nlm.nih.gov/34591551/)  **2021** | **✓** |  |  |  | **346** |  |  | **MH**  **FF/PS** | **-Self-Report Delinquency scale  -Financial strain scale -Protective parenting interactions scale** | **- ProSAAF buffered the negative impact of financial strain on parenting. - Improved protective parenting interactions (b = .3965, p = .0243). - Significant indirect effect of youth-reported financial strain on increases in youth conduct problems through decreases in protective parenting interactions in the intervention group.** | **- Specific demographics may not generalize. - Self-reported youth conduct. - Control did not get personalized attention.** |
| **19** | [**Kirby**](https://pubmed.ncbi.nlm.nih.gov/34781348/)  **2021** |  | **✓** |  |  | **22** |  |  | **MH**  **FF/PS** | **-Perceived Stress Scale (PSS-10)  -Transition Preparation Activities Measure (T-PAM) -Family Empowerment Scale (FES) -Adulthood Expectations Questionnaire (AEQ)** | **- No significant change in parents' coping and stress from pre-intervention to 1-month follow-up (p = .932). - Significant increase in parent and youth transition activities from pre-intervention to post-intervention (p < .001) and 1-month follow-up (p < .05). - Significant increase in parents' self-efficacy (no p-value reported), expectations for their child's future (p < .01), and sense of control over youth outcomes (p < .01) from pre-intervention to 1-month follow-up. - Increased youth self-care skills.** | **- Small sample size. - Demographically homogeneous. - Nonrandomized single group.** |
| **20** | [**Rushovich**](https://www.sciencedirect.com/science/article/pii/S0190740921000074)  **2021** |  |  | **✓** |  | **85** | **155** | **93** | **MH**    **FF/PS** | **-Mini Child and Adolescent Needs and Strengths Survey (Mini-CANS) -Adverse Childhood Experiences (ACEs) Survey  -Protective Factors Survey (PFS) -Mini Child and Adolescent Needs and Strengths Survey (Mini-CANS)** | **- Medium negative effect size for discipline (d = -0.704). - Small negative effect size for child misbehavior (d = -0.238). - Small positive effect sizes for improvements in social support (d = 0.364) and concrete support (d = 0.317) in the treatment group. - Small negative effect size for nurturing and attachment (d = -0.483) in the treatment group.** | **- Small sample size due to little recruitment. - Lack of community resources available for families.** |
| **21** | [**Thulin**](https://journals.sagepub.com/doi/10.1177/1049731519843352)  **2020** |  | **✓** |  |  |  | **764** |  | **MH**  **FF/PS** | **-Trauma Symptom Checklist for Children (TSCC)  -Alabama Parenting Questionnaire-C (APQ-C)** | **- Significant reduction in children's PTSD/trauma symptoms (p<.05); no significant differences in depression and anxiety. - Significant decrease in parental use of corporal punishment after treatment (large effect size, d = 1.12, p = .000); remaining effect at 6-month follow-up (large effect size, d = 1.16, p = .00).** | **- Lack of treatment-as-usual comparison group. - Significant difference in mean age between clinical and non-clinical groups. - Limited knowledge on how new parenting skills are maintained long-term beyond 6-month follow-up. - Five children (two families) reported renewed violence during treatment.** |
| **23** | **[Swendeman 2020](https://pubmed.ncbi.nlm.nih.gov/32574148/)** |  |  |  | **✓** |  | **15** |  | **MH**  **FF/PS** | **-Positive and Negative Affect Schedule (PANAS) scale  -Stattin and Kerr parental monitoring questionnaire -Alabama Parenting Questionnaire -Issues Checklist -Conflict Tactics Scale -Network of Relationships Inventory** | **- Reported positive behavior changes. - Increased participants' awareness of family functioning and led to some self-reported positive behavior changes in decision-making about child input, parental monitoring, quantity and quality of time spent together as a family, parent-child communication (including staying calm during conflicts, giving more praise and less criticism), self-regulation of stress and conflict, parental discipline with a greater focus on positive reinforcement.** | **- Small sample size of families. - Recruitment setting attracts families already interested in improving family functioning. - Smartphone app don't passively monitor phone usage. - Mobile phone usage has adverse effects as well as positive.** |
| **24** | [**Danielson**](https://pubmed.ncbi.nlm.nih.gov/32022827/)  **2020** | **✓** |  |  |  |  | **124** |  | **MH+SU**  **FF/PS** | **-UCLA PTSD Reaction Index for DSM-IV (UCLA-PTSD-RI)  -No specific measurements/tools reported** | **- Significant reductions in PTSD symptoms within groups for RRFT from baseline to months 3 (β = −9.25), 6 (β = −16.63), 12 (β = −17.51), and 18 (β = −19.02) and for TAU from baseline to months 3 (β = −9.62), 6 (β = −13.73), 12 (β = −15.53), and 18 (β = −13.88); no between-group differences observed. - Significant reductions in substance use days from Baseline to month 12 (event rate [ER], 0.28) and month 18 (ER, 0.10). - No outcome for family functioning or parenting skills.** | **- Study sample was primarily female (87.1%). - Unequal length of treatment for the intervention versus TAU.** |
| **27** | **[Holmqvist Larsson 2020](https://pubmed.ncbi.nlm.nih.gov/31419914/)** |  | **✓** |  |  |  | **20** | **21** | **MH**  **FF/PS** | **-Difficulties in Emotion Regulation Scale (DERS) -Toronto Alexithymia Scale (TAS-20) -Levels of Emotional Awareness Scale for Children (LEAS-C) -Beck's Anxiety Inventory (BAI) -Montgomery Åsberg Depression Rating Scale, Self-Report version (MADRS-S)  -Oral feedback** | **- Significant improvement in adolescent emotion regulation (d=0.63, p=.01) and awareness of emotions (d=0.55, p=.02); significant decrease in alexithymia (d=0.57, p=.02). - Significant improvement in parents' emotion regulation (d=0.47, p=.04). - No changes in depression or anxiety for adolescents following the skills training. - Report of gaining a better understanding of their adolescent's emotions, and mutual language for communicating about emotions by parents.** | **- Self-reported measures. - No control group. - No long follow-up. - All female participants. - No random sampling.** |
| **28** | [**McCullough 2019**](https://pubmed.ncbi.nlm.nih.gov/31525563/) |  | **✓** |  |  |  | **54** |  | **MH**  **FF/PS** | **-The Child Behavior Checklist (CBCL) -Assessment Checklist for Children (ACC) -Assessment Checklist for Adolescents (ACA) -Behavioral Rating Inventory of Executive Function (BRIEF)  -Structured parent interviews** | **- Significant reductions in externalizing symptoms (small effect size, Cohen’s D = .025, p = .025), improved affect regulation, behavioral regulation (moderate effect size, Cohen’s d = .435, p = .000), and mental health difficulties (small effect size, Cohen’s D = .212, p = .020), less likely to receive additional mental health diagnoses or be prescribed psychiatric medications in the intervention group compared to the control group (control: 61.9%, intervention: 18.5%, p = .002), and less criminal justice involvement for NPP vs controls.  - Improved parent-child, sibling, and peer relationship quality (medium effect size, p = .005), small improvements in executive functioning (small effect size, Cohen’s d = .147, p = .008), lower placement disruptions (p = .025), and parental separation (p = .002), indicating better family stability.** | **- Small sample size. - Higher mental health diagnoses in the control group.** |
| **30** | [**Colegrove**](https://www.tandfonline.com/doi/full/10.1080/08098131.2019.1616807)  **2019** | **✓** |  |  |  | **26** | **26** | **26** | **MH**  **FF/PS** | **-Childhood Trauma Questionnaire (CTQ) -Brief Betrayal Trauma Questionnaire (BBTS)  -Conflict Behavior Questionnaire (CBQ) -Assessment of Volume and Tempo (AVT) -Assessment of Responsiveness, Reactivity and Turn-taking (ARRT)** | **- More emotional regulation in music tasks after intervention, lower volume and tempo during conflicts, suggesting better emotion regulation (p < .05), and greater consistency in tempo during conflicts, maintaining regulation. - Significant reduction in parent-adolescent conflict and parent reactivity on self-report measures and significant increase in parent emotional responsiveness.** | **- Small sample size. - Type 1 error from no Bonferroni corrections. - Possible bias from author involvement. - Self-reported conflict and family measures. - Possible inaccuracy from adapted measures.** |
| **31** | [**Wijana**](https://pubmed.ncbi.nlm.nih.gov/30477463/)  **2018** |  | **✓** |  |  | **49** | **49** |  | **MH**  **FF/PS** | **-Deliberate Self-Harm Inventory (DSHI-9r) -Youth Self-Report (YSR) -Perceived Stress Scale (PSS-10) -Emotion Regulation Questionnaire (ERQ) -Hospital Anxiety and Depression Scale (HADS) -Child Behavior Checklist (CBCL) -Perceived Stress Scale (PSS-10)  -Questions About Family Members (QAFM)** | **- Significant reductions in adolescents' depression (p = .001), perceived stress (d = 0.54, p = .002), anxiety (d = 0.57, p = .001), internalized symptoms (d = 0.67, p = .001), a 46% decreases in the rate of self-harm behavior in adolescent s (d= 0.54, p= .001) from pre- to post-treatment; significant improvement on the cognitive reappraisal subscale (d = 0.52, p = .002). - Significant reductions in parents' stress levels (mothers: d = 1.02, p = .0001; fathers: d = 0.98, p = .0001); adolescents' perceived criticism from mothers (d = 0.87, p = .001) and fathers (d = 0.42, p = .02) from post-treatment to follow-ups; mothers' critical remarks towards adolescents (d = 0.69, p = .0001) from pre- to post-treatment; mothers' emotional over-involvement (d = 0.69, p = .0001).** | **- Lack of control group. - Small sample size. - Self-reported data. - Short follow-up period. - Sample is predominantly female (85.7%) and limits generalizability of findings.** |
| **32** | [**VanderWesthuizen 2018**](https://pubmed.ncbi.nlm.nih.gov/30236037/) |  | **✓** |  |  | **2** |  |  | **MH**  **FF/PS** | **-DSM-5 Self-Rated Level 1 Cross-Cutting Symptom Measure - Adults -DSM-5 Self-Rated Level 1 Cross-Cutting Symptom Measure - Child Age 11-17 -Children's Global Assessment Scale (C-GAS)  -World Health Organization Disability Assessment Schedule 2.0 (WHODAS)** | **- Adolescents A and B improved in depression, anxiety, irritability, and checking behavior. - Mothers A and B improved in depression, anger, sleep, smoking, homelessness, communication, and daily activities.** | **- Sample size was too small to obtain any significant statistical results. - Relevant process measures were assessed at the level of pre- and post-intervention only.** |
| **33** | [**Gill**](https://link.springer.com/article/10.1007/s10560-017-0510-8)  [**2018**](https://link.springer.com/article/10.1007/s10560-017-0510-8) |  | **✓** |  |  | **12** | **12** | **12** | **MH**  **FF/PS** | **-Difficulties in Emotion Regulation Scale (DERS) -Depression, Anxiety, Stress Scale (DASS-21)  -Evaluation Questionnaire—Adolescent/Parent Versions** | **-Significant improvements in adolescents' anxiety (Cohen's d=0.89, p=0.02, ), stress (Cohen's d=0.83, p=0.01), overall emotion dysregulation (p=0.02), and no statistically significant improvement in depression (Cohen's d=0.73, p=0.19). - No family functioning and parenting skills outcomes.** | **- Small sample size. - No control group. - Improvements could be due to external activities. - Self-selection bias towards more engaged families.** |
